# Immunomodulatory Mechanisms of Rehmanniae Radix Praeparata–Achyranthes Root–Chinese Angelica Root Combination in Nontraumatic Osteonecrosis of the Femoral Head: A Comprehensive Network Pharmacology and Molecular Docking Study Focusing on Immunological Pathways

**DOI:** 10.1155/mi/2808908

**Published:** 2025-12-20

**Authors:** Xin Li, Liqi Ng, Caiying Liu, Leilei Qin, Pengcheng Xiao, Chaozong Liu, Yusong Liu, Qiuping Zhang, Wei Huang, Yu Zhou

**Affiliations:** ^1^ Pharmacy Department, Orthopedic Hospital, Chongqing University of Chinese Medicine, Chongqing Orthopedic Hospital of Traditional Chinese Medicine, Chongqing, 400012, China; ^2^ Institute of Orthopaedic and Musculoskeletal Science, University College London, Royal National Orthopaedic Hospital, Stanmore, London, HA7 4LP, UK, nhs.uk; ^3^ Department of Orthopaedic Surgery, The First Affiliated Hospital of Chongqing Medical University, Chongqing, 400016, China, cqmu.edu.cn; ^4^ Chongqing Municipal Health Commission Key Laboratory of Musculoskeletal Regeneration and Translational Medicine, Chongqing, 400016, China; ^5^ Orthopaedic Research Laboratory of Chongqing Medical University, Chongqing, 400016, China; ^6^ Postdoctoral Research Workstation, Orthopedic Hospital, Chongqing University of Chinese Medicine, Chongqing Orthopedic Hospital of Traditional Chinese Medicine, Chongqing, 400012, China

**Keywords:** data mining, immunomodulation, machine learning, nontraumatic osteonecrosis of the femoral head, UHPLC-Q-TOF-MS^E^

## Abstract

**Objective:**

Nontraumatic osteonecrosis of the femoral head (NONFH) is a devastating condition characterised by immune dysregulation and sterile inflammation, which are increasingly acknowledged as central pathogenic mechanisms. This study aims to identify the core herbal combination for NONFH and systematically explore its immunomodulatory effects and underlying pharmacological mechanisms, with a focus on immune system interactions.

**Methods:**

The core combination ‘Rehmanniae radix praeparata (SDH)‐achyranthes root (NX)‐Chinese angelica root (DG)’ (SND) was identified via data mining of clinical literature using the Traditional Chinese Medicine Inheritance Support System (TCMISS) V2.5 platform. Its chemical constituents were characterised by ultrahigh performance liquid chromatography‐quadrupole time‐of‐flight mass spectrometry (UHPLC‐Q‐TOF‐MS^E^) technology, yielding 127 identified compounds, 47 of which were selected as bioactive components based on drug‐likeness screening. The potential molecular targets of SND and NONFH were predicted and intersected, with a focus on immune‐related targets. Subsequent comprehensive enrichment analysis was performed, emphasising immune pathway involvement, along with immune infiltration profiling. A support vector machine (SVM) model was constructed to identify key immune‐related targets, and interactions were validated via molecular docking and molecular dynamics (MD) simulations.

**Results:**

SND shared 46 candidate targets with NONFH. Enrichment analysis revealed these targets were significantly enriched in immune‐inflammatory pathways, especially those related to immune cell activation and regulation. Notably, pathways involved in neutrophil extracellular trap (NET) formation and other innate immune responses were prominent. Machine learning identified five key targets: ACP1, NDUFAF3, haematopoietic cell kinase (HCK), CXCR2 and platelet‐activating factor receptor (PTAFR)—all of which play critical roles in the modulation, signalling and activation of immune cells, particularly neutrophils and macrophages. Subsequent immune infiltration analysis demonstrated a strong correlation between these key targets (e.g. HCK, CXCR2 and PTAFR) and neutrophil abundance in NONFH. Molecular docking (MD) and molecular dynamics simulations (MDS). MD confirmed stable binding between the active components and these targets, with the HCK‐chrysophanic acid complex exhibiting the strongest affinity (binding energy: −8.7 kcal/mol).

**Conclusion:**

Our integrated analysis suggests that SND alleviates NONFH primarily through multi‐target immunomodulation, explicitly involving suppression of NET formation and regulation of immune cell activity, especially neutrophils and osteoclasts. This study presents a novel, immunologically explicit hypothesis for the mechanism of SND against NONFH, providing a solid theoretical foundation for future experimental validation and clinical application.

## 1. Introduction

Osteonecrosis of the femoral head (ONFH) is a common, progressive and highly disabling refractory disease in the field of orthopaedics. It primarily results from hypoxia and ischaemia within the femoral head, leading to cartilage destruction, local collapse and ultimately progressing to joint space narrowing and restricted joint mobility, resulting in hip dysfunction [1, 2]. Epidemiological data indicate that approximately 200,000–300,000 new cases are diagnosed globally each year, with a rising trend observed annually [3]. In terms of geographical distribution, regions in Asia, such as China, Japan and South Korea, exhibit relatively higher incidence rates, particularly among patients undergoing long‐term steroid therapy [4–6]. In Europe and the Americas, due to the widespread consumption of alcohol and the use of steroids, the incidence is also relatively high [7]. From a demographic perspective, male patients significantly outnumber females, with a male‐to‐female ratio of approximately 3:1. In terms of age distribution, the disease predominantly affects individuals aged between 30 and 50 years [8, 9]; however, the specific age of onset may vary depending on the aetiology. Notably, nontraumatic ONFH (NONFH) accounts for over 70% of all ONFH cases [10]. NONFH is primarily associated with a series of risk factors, including long‐term use of steroids, hormonal medications, autoimmune diseases and excessive alcohol consumption [3, 11]. Due to the complexity of the aetiological mechanisms underlying NONFH, there are currently no effective approved drugs available for the treatment of ONFH. Surgical interventions, including hip replacement, remain the mainstream approach in modern medicine. However, the efficacy of surgical treatments varies and is heavily dependent on the surgeon’s experience, while also posing risks such as economic burden, revision surgery and various complications [2, 12, 13].

Emerging evidence highlights the crucial role of immune inflammation in the pathogenesis of NONFH [14, 15]. Exposure to steroids and alcohol can trigger neutrophil activation and the release of pro‐inflammatory mediators, which can cause vascular endothelial injury, intraosseous coagulation and eventually osteonecrosis [16, 17]. Recent research particularly underscores the role of neutrophil extracellular traps (NETs) in contributing to vascular dysfunction in ONFH [18, 19].

In recent years, traditional Chinese medicine (TCM) has been widely used as an alternative therapy for ONFH due to its proven efficacy and safety [20–23]. Mechanistic studies have shown that TCM can alleviate the progression of ONFH by regulating bone metabolism, oxidative stress and lipid metabolism [23–25]. However, current research in this area primarily focuses on the herbal components or formulae with proven efficacy, lacking in‐depth exploration of formulation principles and mechanisms of action [26]. In addition, due to the high variability and complexity of clinical TCM practice, there is a need to continuously optimise drug treatment regimens and systematically investigate the formulation principles and mechanisms of action of TCM formulae.

Data mining techniques can analyse large clinical prescription datasets using algorithms to identify core drugs and uncover, hidden valuable information [27, 28]. Ultrahigh performance liquid chromatography‐quadrupole time‐of‐flight mass spectrometry (UHPLC‐Q‐TOF‐MS^E^) is a high‐resolution, highly sensitive analytical tool with structural characterisation capabilities, enabling comprehensive identification of components in drug combinations obtained through data mining, which helps elucidate their pharmacological basis [29]. Network pharmacology employs systems biology, bioinformatics and network science methods to construct visual ‘drug‐target‐disease’ networks, revealing the complex mechanisms of TCM in treating diseases [30, 31]. However, traditional network pharmacology has low result accuracy, and integrating UHPLC‐Q‐TOF‐MS^E^ identified components as candidates with network pharmacology and bioinformatics methods can effectively reduce false positive rates. Molecular docking (MD) uses computational simulations based on the lock‐and‐key principle between drug molecules and proteins to analyse their interactions. In contrast, molecular dynamics simulations (MDS) can provide further insight into these interactions at the molecular level [32, 33].

Therefore, this study first utilises the TCM Inheritance Support System (TCMISS) V2.5 platform to screen the highest‐confidence herbal combination for treating NONFH in TCM UHPLC‐Q‐TOF‐MS^E^ technology, and the UNIFI platform is employed to identify chemical components in the combination and summarise their fragmentation patterns. Subsequently, the active components of the combination are further screened based on drug similarity principles, and comprehensive bioinformatics and target pharmacology studies are conducted, including WGCNA construction, target organ and GO/KEGG enrichment analysis, and machine learning combined with SHAP to identify key targets. This aims to explore the core components, key targets and molecular mechanisms of the combination in treating NONFH. Finally, MD and MDS techniques are used to investigate the binding ability between core components and key targets. The results of this study will help further uncover the potential mechanisms of TCM in treating NONFH, providing a reference for subsequent clinical treatment and in‐depth research.

## 2. Materials and Methods

### 2.1. Literature Data Mining

#### 2.1.1. Literature Sources

Search terms including ‘Osteonecrosis of the femoral head’, ‘ONFH’, ‘Traditional Chinese Medicine’, ‘Herbal Medicine’ and ‘Integration of Chinese and Western Medicine’ were used to retrieve relevant literature from the following databases: CNKI, VIP, PubMed, WanFang Database, Web of Science and Cochrane Library. The search time span ranged from the establishment of each database to 13 September, 2024. After deduplication and organisation, a total of 10,093 retrieved literature were reduced to 590 unique articles. Among these, 53 studies were selected based on inclusion criteria, including clinical controlled trials and self‐controlled trials. Two independent researchers were responsible for data extraction and entry.

#### 2.1.2. Inclusion Criteria

The following criteria were applied: ① Participants must be clinical patients meeting the diagnostic criteria for ONFH according to both Chinese and Western medicine standards. ② All cases must be NONFH, including alcohol‐related and steroid‐induced cases. ③ The intervention group must receive only oral Chinese herbal formulae. ④ The study design and statistical methods must be scientifically valid. ⑤ Only one representative study is included for cases of duplicate or same‐formula literature. ⑥ The study must explicitly specify the names and dosages of Chinese herbal medications. ⑦ The treatment efficacy rate must be ≥80%.

#### 2.1.3. Exclusion Criteria

The following criteria were applied: ① Dissertations, reviews, medical case reports, individual case studies and so on. ② Basic experimental studies, meta‐analyses and nursing‐related studies. ③ Lack of clinical data or study results not meeting the inclusion criteria. ④ Patients with comorbidities or severe complications. ⑤ Patients receiving concurrent therapies such as acupuncture, massage, tuina, Western medicine, surgery or nursing interventions. ⑥ Cases of ONFH caused by trauma or fractures.

#### 2.1.4. Normalisation of TCM

In this study, we refer to the 2020 edition of the Chinese Pharmacopoeia to standardise the names of Chinese medicines used in prescriptions, thereby avoiding the influence of different concoctions or aliases on the final analysis results [27].

#### 2.1.5. Prescription Entry and Data Analysis

Two staff members entered the eligible prescriptions into the TCMSS V2.5 platform. Within the ‘Platform Management’ and ‘Formulation Management’ modules, a ‘NONFH Formulation Database’ was established. After data entry, the accuracy of the information was verified. Subsequently, in the ‘Data Analysis’ and ‘Statistical Reports’ modules, the frequency of Chinese herb usage, their properties (taste and meridian tropism), formulation patterns, core combinations and potential new formulations were analysed. The drug combination mode with the highest confidence level was selected for further research.

### 2.2. In Vitro Compositional Identification of Rehmanniae Radix Praeparata (SDH)‐Achyranthes Root (NX)‐Chinese Angelica Root (DG) (SND)

#### 2.2.1. Instruments and Reagents

Waters Acquity H‐Class UHPLC type ultra performance liquid chromatography (Waters Corporation, USA); SYNAPT XS quadrupole time of flight mass spectrometer (Waters Corporation, USA); ME204/02 type electronic balance (Shanghai METTLER TOLED Company); KS‐2200DE liquid crystal ultrasonic cleaner (Kunshan Jielimei Ultrasonic Instrument Co., Ltd.).

Rehmanniae Radix Praeparata, Achyranthes Root, Chinese Angelica Root were purchased from Orthopaedic Hospital Affiliated to Chongqing university of Chinese Medicine; *β*‐Ecdysterone, cyasterone, oleanolic acid, ferulic acid and chlorogenic acid were all purchased from the China Academy of Food and Drug Administration, and the purity was ≥98%; acetonitrile (acetate), acetonitrite (acetone) and chloroform were all purchased from the China Academy of Food and Drug Administration, the purity was ≥98%. 98%; acetonitrile (chromatographic purity, Merck, Germany); formic acid (chromatographic purity, Aladdin, Shanghai); ultrapure water prepared by Milli‐Q system (Millipore, USA); leucine enkephalin and sodium formate (Waters, USA).

#### 2.2.2. Detection Conditions

Chromatographic conditions: ACQUITY UHPLC BEH C18 column (2.1 mm × 100 mm, 1.7 μm); mobile phase 0.1% formic acid in water (A)‐0.1% formic acid in acetonitrile (B), positive/negative ion mode gradient elution 0–4 min 20% B; 4–15 min 20%–35% B; 15–30 min 35%–85% B; 30–35 min 85%–95% B; 35–45 min 95%–95% B; 45–50 min 95%–20% B; at a flow rate of 0.8 mL/min, with a column temperature of 40°C and an injection volume of 10 μL.

Mass spectrometry conditions: electrospray ESI ion source, positive and negative ion modes, MSE full scan mode, scan time 1.0 s, mass scan range *m*/*z*: 100–2000; ion source temperature 120°C, desolventisation temperature 450°C, cone pore gas (N_2_) and desolventisation gas (N_2_) flow rates of 50 and 800 L/h in positive and negative ion modes; capillary voltage and cone pore voltage of 3.0 and 40 kV in positive ion mode, 2 and 40 kV in negative ion mode. The capillary voltage and cone pore voltage were 3.0 and 40 kV in the positive ion mode and 2 and 40 kV in the negative ion mode. The collision energy was used in MS_E_, with a low collision energy of 4 V and a high collision energy of 30–45 V. The leucine enkephalin (*m*/*z* 556.2771 in the positive ion mode and 554.2615 in the negative ion mode) was calibrated using sodium formate calibration of the mass spectrometer (real‐time mass).

#### 2.2.3. Preparation of Control and Test Solution

Preparation of control solution: weigh 2 mg each of *β*‐ecdysterone, cyasterone, oleanolic acid, ferulic acid and chlorogenic acid, add 1 mL of methanol to dissolve, take 100 μL of each control, add 500 μL of methanol, add 1 mL, filter through a 0.22 μm microporous membrane, transfer to liquid phase vials and stand by.

Preparation of test solution: 18 g of rehmanniae radix praeparata, 15 g of achyranthis bidentatae radix, 15 g of angelicae sinensis radix, crushed into coarse particles, add 10 times distilled water, soak for 1 h, the first reflux extraction for 2 h, filtration; residue add eight times water, the second reflux extraction for 1.5 h, filtration. Combine the two filtrates, 60–70 °C concentrated under reduced pressure to a thick paste; the concentrate was placed in a lyophiliser to lyophilise; the resulting lyophilised powder was stored in a cool, dry place. Take 0.1 g of SND lyophilised powder and dissolve it in 5 mL of methanol, centrifuge it at 10,000 rpm for 10 min, take 1 mL of the supernatant, pass it through a 0.22 μm microporous filter membrane and transfer it to a liquid phase bottle for spare use.

#### 2.2.4. Self‐Constructed Sub‐Database

With the help of relevant literature and online databases such as PubChem (https://pubchem.ncbi.nlm.nih.gov), the chemical composition information of each flavour of SND herbal medicine was searched, and a database of SND herbal medicine compositions was established and imported into the UNIFI platform.

### 2.3. Identification of the Main Types of SND Compounds and Analysis of Cleavage Patterns

By reviewing the relevant literature, combining the information on mass spectrometry fragments and referring to the information on control products, the cleavage patterns of the main chemical components of SND were deduced and analysed, which provided a reliable theoretical basis for the subsequent study of pharmacodynamic substances and the formulation of quality standards.

### 2.4. Targeted Pharmacological Analysis

#### 2.4.1. Drug Similarity Analysis

The identified chemical components were entered into the PubChem database to obtain the Canonical SMILES sequence numbers, followed by further screening of the active ingredients of SND in the SwissADME (http://www.swissadme.ch/) platform [34]. First, gastrointestinal absorption ‘High’ was used as a criterion to ensure the bioavailability of the active ingredient; secondly, drug similarity screening rules (Egan, Lipinski, Veber, Ghose, Muegge) were used as a criterion to ensure that the active ingredient could be screened only if there were at least two ‘YES’ to be included in the active ingredient category.

#### 2.4.2. Obtaining SND Prediction Targets

The active ingredients meeting the screening criteria under ‘1.4.1’ were used as candidate compounds, and were searched in the Swiss Target Prediction database (http://www.swisstargetprediction.ch) [35], the SuperPred database (https://prediction.charite.de/) and the SEA database (https://sea.bkslab.org/) to predict their relevant targets. Components without targets were removed, and the predicted targets obtained above were summarised and de‐emphasised to establish the predicted target set of the SND compound.

#### 2.4.3. Establishing the NONFH Target Database

Searches were conducted in the Gene Expression Omnibus (GEO; www.ncbi.nlm.nih.gov/geo/) to retrieve mRNA expression profiles (GSE123568) between NONFH and normal samples. Differential genes (DEGs) between NONFH and normal samples were identified with screening thresholds of |logFC| > 0.05 and *p* < 0.05, and a volcano plot of DEGs was obtained. The ‘WGCNA’ R package was utilised to identify co‐expression modules. To ensure accuracy, the top 25% of genes with the highest variability were applied to subsequent analyses. The optimal soft threshold was selected to construct the weighted adjacency matrix, which was subsequently converted into a topological overlap matrix (TOM). The minimum module size was set to 100, and TOM dissimilarity metrics (1‐TOM) based on hierarchical clustering tree algorithms were employed to obtain modules.

#### 2.4.4. Intersection Target Screening and GO/KEGG, Target Organ Enrichment Analysis

The microbioinformatics online platform (http://www.bioinformatics.com.cn/) [36] was used to clarify the overlap between SND predicted targets and NONFH disease targets, obtaining ‘SND‐NONFH’ intersection targets. Given the unclear metabolic processes of toxicity candidate targets in the human body and their potential involvement in multiple organs, tissues and immune cells in the pathogenesis of NONFH, the intersection targets were entered into the DAVID online platform (https://davidbioinformatics.nih.gov/) for enrichment analysis [37]. This aimed to explore the enrichment of intersection targets in various human tissues and organs. In addition, GO/KEGG enrichment analysis was conducted on this platform, with *Homo sapiens* as the study target. GO enrichment analysis primarily included biological processes (BP), cellular components (CC) and molecular functions (MF). The top 5 most significant GO items and the top 20 KEGG pathways were screened and visualised using the microbioinformatics online platform. The results were presented in bubble charts and histograms.

#### 2.4.5. Constructing Machine Learning Models and SHAP Analysis for Identifying Key Targets

To further identify characteristic genes associated with ‘SND‐NONFH’, this study constructed eight machine learning methods (GLM, support vector machine [SVM], glmboost, RF, XGB, KNN, NNET and PLS) to better predict and identify candidate targets. The SHAP (SHapley Additive exPlanations) method enables the interpretation and visualisation of the contribution of feature toxicological targets to the performance of machine learning models [38]. Conducting SHAP analysis on the best‐performing machine learning model aids in identifying genes that significantly influence disease progression [39]. We utilised the SHAP library to obtain SHAP values for candidate targets, with results presented as histograms. The top genes had a greater impact on the model compared to the bottom genes.

#### 2.4.6. Immunological Infiltration Analysis

The CIBERSORT algorithm was employed to analyse gene expression data across 22 immune cell types between NONFH and healthy samples, assessing the relative abundance of each immune cell type in individual samples. A *p* < 0.05 indicated significant differences. To further validate the association between key targets of NONFH and SND with characteristics of related immune cells, the ‘corrplot’ package was used to analyse the relative percentage correlation coefficients between them.

#### 2.4.7. Validation of Key Targets

The expression levels of key targets were analysed in both the NONFH group and the control group. Subsequently, the relationships among these genes were investigated.

#### 2.4.8. Molecular Docking and MD Simulation Validation

The results of MD can provide evidence for the binding activity between Chinese herbal medicine (CHM) active components and important target proteins to a certain extent, serving as auxiliary validation for the aforementioned target pharmacological results. In this study, key targets were used as receptor macromolecules, and their corresponding CHM active compounds were used as ligand micromolecules for molecular docking validation. The 3D structures of the receptors were sourced from the PDB database (http://www.rcsb.org/); ligand structures in SDF format were obtained from the PubChem database (https://pubchem.ncbi.nlm.nih.gov/), and subsequently converted to PDB format using OpenBabel‐3.1.1 software. PyMOL software was used to remove small molecule ligands, water molecules and other components from the receptor structures. The AutoDockTools‐1.5.7 software was used for hydrogenation and charge calculation of the receptor proteins, completing the MD analysis. PyMOL software was then used to visualise the conformation with the lowest binding energy.

To further validate the stability of the MD results, MD simulations were conducted to simulate the dynamic interaction process between the protein and the compound, identifying potential low‐energy conformations and enhancing the rationality and scientific nature of drug design. The GROMACS software was used for MD simulations. The protein system utilised the CHARMM 36 force field, along with the TIP3P water model and a dodecahedron‐shaped water box. The distance between the complex and the box edge was at least 1.0 nm. NVT equilibration was performed for 100 ps, followed by NPT equilibration for 100 ps. Various parameters were calculated to analyse the binding mode of the protein–compound complex and the changes in protein compactness with and without the ligand. These parameters included root‐mean‐square deviation (RMSD), root‐mean‐square fluctuation (RMSF), radius of gyration (Rg) and hydrogen bonds. RMSD represents the sum of atomic deviations between a given time frame and the target conformation, serving as a critical foundation for assessing system stability and providing detailed structural information for each system’s conformational stability [40]. The Rg radius curve represents the root‐mean‐square distance of protein atoms from the rotational axis, indicating the general compactness of the protein’s conformation [41]. Gibbs free energy landscapes (FELs) were plotted based on the RMSD and gyration values of the complex to obtain the lowest energy conformation of the docking results.

## 3. Results

### 3.1. Literature Mining Results

#### 3.1.1. Frequency of CHM Use

A total of 53 studies were included based on the inclusion and exclusion criteria, from which 53 prescriptions were extracted, involving 122 CHMs. The frequency of occurrence for each herb was statistically analysed, with 22 herbs appearing ≥10 times. The top five herbs were Angelicae Sinensis Radix, Glycyrrhizae Radix Et Rhizoma, Achyranthis Bidentatae Radix, Drynariae Rhizoma and Chuanxiong Rhizoma, as detailed in Table 1.

**Table 1:** TCM treatment of CHMs with NONFH frequency ≥10.

Rank	CHM	Frequency	Average dose (g)	Rank	CHM	Frequency	Average dose (g)
1	Angelicae sinensis radix	38	14.52	12	Eucommiae cortex	18	15.94
2	Glycyrrhizae radix Et rhizoma	35	7.97	13	Paeoniae radix alba	17	18.68
3	Achyranthis bidentatae radix	33	14.97	14	Astragali radix	17	26.75
4	Drynariae rhizoma	29	14.74	15	Spatholobi caulis	16	17.38
5	Chuanxiong rhizoma	25	14.00	16	Angelicae pubescentis radix	15	11.78
6	Atractylodis macrocephalae rhizoma	24	13.48	17	Paeoniae radix rubra	15	12.43
7	Salviae miltiorrhizae eadix Et rhizoma	24	24.68	18	Notoginseng radix Et rhizoma	14	9.62
8	Dipsaci radix	23	13.26	19	Corydalis rhizoma	11	14.90
9	Poria	23	16.04	20	Cervi cornus colla	10	11.50
10	Rehmanniae radix praeparata	23	17.45	21	Carthami flos	10	10.78
11	Epimedii folium	20	13.18	22	Eupolyphaga steleophaga	10	9.10

#### 3.1.2. Statistical Analysis of the Flavours and Attributes Meridians of CHMs

The four qi (四气) and five flavours (五味) of 122 CHM were counted (Table 2 and Figure 1A,B). The frequencies of warm, neutral, cold, heat and cool CHM were 410, 175, 123, 23 and 9 in that order; and the frequencies of sweet, bitter, pungent, sour, salty and astringent CHM were 402, 345, 284, 67, 45 and 23 in that order. Statistics on the CHM attributed to the meridians (归经) (Figure 1C) showed that the top five CHM were attributed to the liver, kidney, spleen, heart and stomach in order; among them, the frequency attributed to the liver meridian could be up to 529, which was the highest among them.

Figure 1Statistical chart of the four qi, five flavours and attributes of CHMs for the treatment of NONFH. (A) the four natures and five flavours of HMs. (B, C) the channel tropism and property‐flavour characteristics of HMs.

**Figure:**
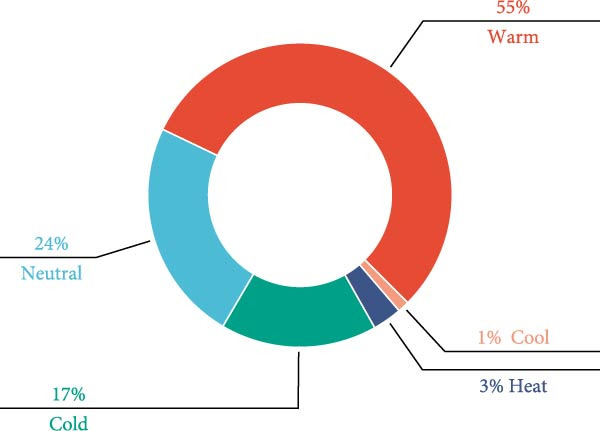
(A)

**Figure:**
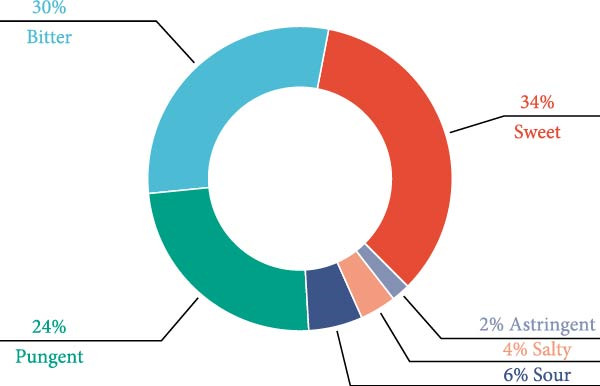
(B)

**Figure:**
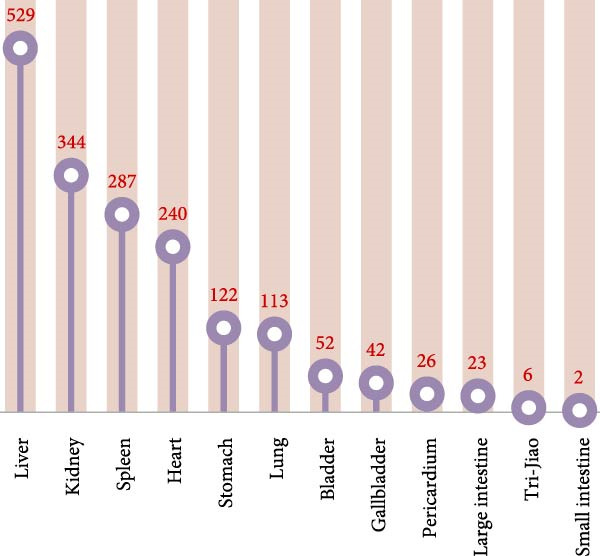
(C)

**Table 2:** Statistics of the four qi and five flavours of CHNs for the treatment of NONFH.

Rank	Four qi	Frequency	Rank	Five flavours	Frequency
1	Warm	410	1	Sweet	402
2	Neutral	175	2	Bitter	345
3	Cold	123	3	Pungent	284
4	Heat	23	4	Sour	67
5	Cool	9	5	Salty	45
—	—	—	6	Astringent	23

#### 3.1.3. Analysis of Core Combinations and New Prescription Combinations

With reference to the number of prescriptions and CHMs, combined with different correlation and penalty parameters, the results were more in line with clinical practice when correlation = 8 and penalty = 3. Through complex system entropy clustering analysis, six groups of two different core combinations were extracted; based on the improved mutual information method, the correlation coefficients between the two CHMs were obtained through the analysis method of complex system entropy clustering, and then, through the cluster analysis, 6 potential new formulas were obtained from the 53 prescriptions (Table S1).

#### 3.1.4. Research on the Law of Formulation Based on Association Rule Analysis

Under the Apriori association rule algorithm, the higher the frequency (i.e., support) of CHM combinations appearing in the selected prescription, the stronger the association between the core drugs. The closer the confidence level (when the probability of the CHM on the left side of ‘→’ and the CHM on the right side appearing at the same time) is to 1, the higher the frequency of the drugs appearing at the same time on both sides of ‘→’. In the module of ‘Formulary pattern’, the support degree was set to 15. The confidence level was set to 0.90, which resulted in a total of 40 pairs of CH pairing patterns with a frequency ≥15, involving 12 CHMs, which were ranked according to the frequency of CHM combinations in ascending order (Table S2). A total of three rules for the use of commonly used CHMs were obtained at the same time (Table 3).

**Table 3:** Association rule analysis of CHMs for treatment of NONFH.

Rank	CHMs association rules	Confidence level
1	Rehmanniae radix praeparata → angelicae sinensis radix	0.913043478
2	Rehmanniae radix praeparata, achyranthis bidentatae radix → angelicae sinensis radix	0.944444444
3	Atractylodis macrocephalae rhizoma, angelicae sinensis radix → glycyrrhizae radix Et rhizoma	0.941176471

As can be seen from Table 3, SND was the most confident pairing mode, so this study focused on this CHMs pairing mode to identify and analyse its specific pharmacodynamic substance basis and mechanism of action.

### 3.2. In Vitro Compositional Analyses of SND Chinese Medicine Pairs

#### 3.2.1. Data Acquisition

The chromatographic and mass spectrometric conditions under 2.2.2.1 were used, and the UHPLC‐Q‐TOF‐MS^E^ technique was used to detect the test solution and the control solution, and to obtain the base peak ion (BPI) chromatograms of the positive and negative ions (Figure 2A,B).

Figure 2BPI plots of SND in positive ion mode (A) and negative ion mode (B).

**Figure:**
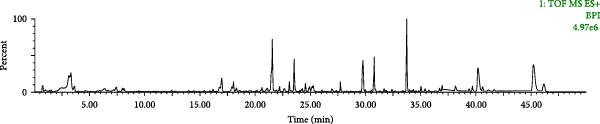
(A)

**Figure:**
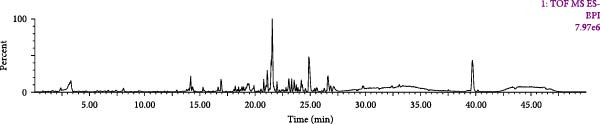
(B)

#### 3.2.2. Self‐Composition Database

With the help of relevant literature and online databases such as PubChem, the chemical composition information of each Chinese medicine in the SND pairs was searched, and the database of Chinese medicine composition in the SND pairs was established and imported into the UNIFI software.

#### 3.2.3. Data Analysis

MassLynx V4.1 software was used to collect the data and quickly match the database in the UNIFI platform, and the compound error screening criterion was <1 × 10^−5^. The compounds were identified by comparing the control mass spectrometry information, secondary mass spectrometry fragmentation ion information, references and mass spectrometry cleavage patterns. A total of 127 compounds were deduced and identified, including 38 terpenoids, 29 organic acids, 11 phthalides, nine phenylethanol glycosides, eight phenylpropanoids, eight flavonoids, eight steroids and 16 other compounds, and the results are shown in Table 4.

**Table 4:** Identification of chemical constituents of SND.

Peak (number)	*t*_R_ (min)	Molecular formula	Ion mode	Found mass (*m*/*z*)	10^−6^ × error	Fragment (*m*/*z*)	Compound	Attribution
P1	0.78	C_12_H_22_O_11_	[M−H]^−^	341.1079	−2.90	Melibiose	221.0662, 179.0533	SDH
P2	0.79	C_6_H_12_O_7_	[M−H]^−^	195.0502	−4.29	Gluconic acid	129.0197	DG, NX
P3	1.16	C_15_H_22_O_10_	[M+HCOO]^−^	407.1180	−3.69	Monomelittoside	199.0620, 179.0533, 161.0454	SDH
P4	1.17	C_6_H_8_O_7_	[M−H]^−^	191.0190	−3.59	Citric acid	173.0064	DG, SDH
P5	1.21	C_6_H_14_N_4_O_2_	[M+H]^+^	175.1190	0.15	Arginine	158.0963, 130.0988, 116.0716	DG, NX
P6	1.22	C_16_H_18_O_9_	[M−H]^−^	353.0871	−3.43	Chlorogenic acid	191.0530, 179.0533, 173.0419, 161.0454, 135.0442	DG
P7	1.25	C_26_H_32_O_13_	[M−H]^−^	551.1723	−8.59	Durantoside Ⅰ	193.0497	SDH
P8	1.28	C_17_H_20_O_9_	[M−H]^−^	367.1030	−1.14	3‐O‐feruloylquinic acid	193.0497, 173.0452, 134.0376	DG
P9	1.30	C_26_H_31_NO_10_	[M+H]^+^	518.2053	6.26	2,4,10‐Trimethoxy‐dihydro‐6H‐ isoquinolino[3,2‐a]isoquinoline‐ 3,11‐diol‐glucoside	177.0552	NX
P10	1.34	C_16_H_20_O_9_	[M+HCOO]^−^	401.1121	7.82	6‐O‐feruloylglucose	265.0745, 193.0497	SDH
P11	1.35	C_36_H_48_O_20_	[M−H]^−^	799.2693	3.31	Jionoside A1	193.0497	SDH
P12	1.40	C_22_H_30_O_13_	[M+HCOO]^−^	547.1664	−0.83	Sibirioside B	377.0928	SDH
P13	1.41	C_31_H_38_O_16_	[M−H]^−^	665.2143	8.41	2^′^‐Acetylacteoside	161.0235	SDH
P14	1.48	C_13_H_14_N_2_O_2_	[M+H]^+^	231.1123	−2.24	1,2,3,4‐Tetrahydroharmane‐3‐carboxylic acid	214.0876, 188.0714, 158.0953	DG
P15	1.49	C_29_H_36_O_15_	[M−H]^−^	623.2019	6.09	Isoacteoside	161.0235	SDH
P16	1.56	C_34_H_28_O_22_	[M−H]^−^	787.0974	−3.20	1,2,3,6‐Tetragalloylglucose	295.0497	DG
P17	1.58	C_8_H_6_O_4_	[M−H]^−^	165.0182	−6.63	Phthalic acid	121.0275	DG
P18	1.67	C_8_H_8_O_4_	[M‐H]^-^	167.0337	−7.67	Vanillic acid	152.0387, 123.0420	NX, DG,SDH
P19	1.89	C_12_H_16_O_8_	[M+HCOO]^−^	333.0834	2.12	5‐GMF	125.0249	SDH
P20	1.90	C_9_H_10_O_4_	[M−H]^−^	181.0506	−0.44	Dihydrocaffeic acid	135.0444	SDH
P21	1.90	C_12_H_16_O_5_	[M−H]^−^	239.0918	−3.03	Senkyunolide R	177.0921	DG
P22	1.92	C_16_H_22_O_10_	[M+HCOO]^−^	419.1217	5.29	Geniposidic acid	211.0641, 193.0484, 149.0583	SDH
P23	1.93	C_27_H_44_O_7_	[M−H]^−^	479.3041	6.68	Ecdysterone	319.1927, 301.0117, 159.0990	NX
P24	1.94	C_37_H_50_O_20_	[M−H]^−^	813.2855	3.96	Jionoside B1	619.2290, 193.0495, 160.0163	SDH
P25	2.25	C_10_H_16_O_3_	[M−H]^−^	183.1027	−0.09	Rehmapicrogenin	139.1123	SDH
P26	2.26	C_10_H_10_O_4_	[M‐H]^-^	193.0484	−8.81	Ferulic acid	179.0533, 149.0583, 135.0442	DG, NX
P27	2.28	C_29_H_44_O_9_	[M+HCOO]^−^	581.2963	−0.77	Sengosterone	499.2682	NX
P28	2.38	C_25_H_24_O_12_	[M−H]^−^	515.1219	4.67	3,4‐Dicaffeoylquinic acid	353.0910, 191.0588	DG
P29	2.38	C_29_H_36_O_15_	[M−H]^−^	623.1992	1.74	Acteoside	461.1659, 161.0248, 135.0443	SDH
P30	2.49	C_31_H_40_O_16_	[M−H]^−^	667.2179	−9.72	Hemiphroside A	193.0495	SDH
P31	2.52	C_28_H_46_O_7_	[M+HCOO]^−^	539.3219	−1.17	Makisterone B	319.1908, 301.1797	NX
P32	2.54	C_9_H_10_O_3_	[M−H]^−^	165.0550	−4.54	Acetovanillone	147.0444	SDH
P33	2.55	C_35_H_46_O_20_	[M+HCOO]^−^	831.2647	9.91	Echinacoside	161.0248	SDH
P34	2.55	C_13_H_14_N_2_O_3_	[M−H]^−^	245.0933	0.70	N‐acetyltryptophan	203.0805, 159.0812, 116.0489	DG
P35	2.73	C_25_H_30_O_13_	[M−H]^−^	537.1628	2.69	6‐O‐feruloylcatalpol	337.0983, 193.0484, 175.0389, 149.0583	SDH
P36	3.14	C_12_H_22_O_11_	[M+HCOO]^−^	387.1135	−2.26	Sucrose	161.0438	NX, SDH
P37	3.17	C_30_H_38_O_15_	[M−H]^−^	637.2132	9.00	Leucosceptoside A	491.1533, 461.1664, 315.1094, 179.0533	SDH
P38	3.31	C_5_H_10_O_5_	[M+HCOO]^−^	195.0506	−2.12	D‐ribose	149.0229	NX
P39	3.31	C_29_H_44_O_8_	[M−H]^−^	519.2971	−0.89	Cyasterone	319.1864, 301.1767, 199.0968	NX
P40	3.40	C_29_H_44_O_9_	[M+HCOO]^−^	581.2965	−0.33	24‐Hydroxycyasterone	535.2909, 499.2642	NX
P41	3.56	C_21_H_34_O_9_	[M−H]^−^	429.2092	−8.79	Jiocarotenoside A1	249.1157, 205.1259	SDH
P42	3.62	C_27_H_42_O_20_	[M−H]^−^	685.2257	8.80	Rehmannioside D	179.0533	SDH
P43	3.81	C_9_H_16_O_4_	[M−H]^−^	187.0963	−6.63	Azelaic acid	169.0880, 125.0966	DG
P44	4.64	C_25_H_32_O_12_	[M−H]^−^	523.1807	−2.73	6‐O‐E‐feruloylajugol	193.0484, 175.0389	SDH
P45	4.78	C_12_H_16_O_5_	[M−H]^−^	239.0907	−7.44	Senkyunolide S	195.1028, 177.0911	DG
P46	5.14	C_14_H_14_O_4_	[M+H]^+^	247.0942	−9.18	Marmesin	225.0493, 169.0688	DG
P47	5.14	C_12_H_14_O_3_	[M+H]^+^	207.1007	−4.41	Senkyunolide F	189.0901, 179.0706	DG
P48	5.71	C_29_H_48_O_7_	[M+HCOO]^−^	553.3385	0.56	Amarasterone B	507.3280, 301.1809	NX
P49	5.73	C_16_H_12_O_4_	[M+H]^+^	269.0802	−2.55	Formononetin	254.0580, 237.0480, 213.0891, 136.0155	DG
P50	5.95	C_18_H_19_NO_4_	[M−H]^-^	312.1248	2.14	N‐trans‐feruloyltyramine	297.1033, 148.0500	NX
P51	5.97	C_41_H_62_O_15_	[M+HCOO]^−^	839.4007	−7.54	28‐Deglucosyl‐achyranthoside C	793.3906	NX
P52	5.97	C_11_H_14_O_3_	[M−H]^−^	193.0870	0.01	Butylparaben	136.0168, 108.0219	DG
P53	6.25	C_31_H_40_O_15_	[M−H]^−^	651.2311	2.48	Martynoside	506.1962, 329.1220	SDH
P54	6.65	C_19_H_21_NO_5_	[M+H]^+^	344.1486	−1.90	N‐feruloyl‐3‐methoxytyramine	177.0547, 145.0275, 117.0547	NX
P55	6.69	C_10_H_8_O_3_	[M+H]^+^	177.0545	−0.85	6‐Methoxycoumarin	149.0604, 145.0291, 117.0340	DG
P56	6.70	C_31_H_40_O_15_	[M−H]^−^	651.2306	1.85	Isomartynoside	475.1767, 175.0385	SDH
P57	7.19	C_15_H_10_O_6_	[M−H]^−^	285.0409	1.69	Kaempferol	271.9315, 229.4565, 185.9460	NX
P58	7.22	C_21_H_20_O_11_	[M−H]^−^	447.0962	6.53	Quercitrin	301.0362, 151.0031	NX
P59	7.75	C_47_H_74_O_18_	[M−H]^−^	925.4800	−0.21	Stipuleanoside R1	763.424	NX
P60	7.98	C_16_H_12_O_5_	[M−H]^−^	283.0604	−2.64	Wogonin	163.7236	NX
P61	8.09	C_4_H_6_O_5_	[M−H]^−^	133.0142	−0.21	Malic acid	115.0038	DG
P62	8.22	C_30_H_46_O_5_	[M+H]^+^	487.3394	−4.91	(3*β*)‐3,19‐Dihydroxy‐2‐oxours‐12‐en‐28‐oic acid	469.3203, 425.3351	NX
P63	9.96	C_41_H_64_O_13_	[M+HCOO]^−^	809.4270	−7.24	Ophiopogonin A	763.424	NX
P64	10.28	C_27_H_30_O_14_	[M−H]^−^	577.1547	−2.78	Rhoifolin	269.0886	NX
P65	11.08	C_16_H_26_O_8_	[M+HCOO]^−^	391.1606	−1.02	Rehmapicroside	179.0580, 165.0913	SDH
P66	12.15	C_18_H_32_O_5_	[M−H]^−^	327.2163	−4.18	(10E,15E)‐9,12,13‐trihydroxyoctadeca‐ 10, 15‐dienoic acid	229.1422, 211.1324	DG
P67	12.19	C_15_H_10_O_5_	[M−H]^−^	269.0459	1.43	Baicalein	251.0583	DG, NX
P68	12.61	C_15_H_10_O_4_	[M+HCOO]^−^	299.0561	−0.12	Chrysophanic acid	209.0487, 225.3369	NX
P69	12.88	C_47_H_74_O_18_	[M+H]^+^	927.4896	−5.66	Niuxi saponin A	455.3503	NX
P70	13.99	C_18_H_34_O_5_	[M−H]^−^	329.2318	−4.61	Pinellic acid	329.2332, 171.1023	NX
P71	14.15	C_20_H_22_O_7_	[M−H]^−^	373.1297	1.12	Epinortrachelogenin	223.0650, 150.0313	NX
P72	14.31	C_53_H_82_O_25_	[M−H]^−^	1117.5162	8.00	Achyranthoside D	955.4910, 793.4293, 731.4335, 631.0873, 455.3485	NX
P73	14.46	C_48_H_76_O_19_	[M+HCOO]^−^	1001.4875	−8.76	Ginsenoside Ro	161.0441	NX
P74	14.62	C_36_H_62_O_9_	[M+HCOO]^−^	683.4356	−2.97	Ginsenoside Rh1	637.432	NX
P75	14.71	C_21_H_28_O_13_	[M+HCOO]^−^	533.1542	5.56	Cistanoside F	251.0544, 161.0275	SDH
P76	14.98	C_48_H_74_O_19_	[M−H]^−^	953.4760	0.89	Achyranthoside E dimethyl ester	791.4177	NX
P77	15.30	C_54_H_86_O_23_	[M−H]^−^	1101.5544	5.21	Niuxi saponin B	455.3534	NX
P78	15.38	C_29_H_36_O_13_	[M−H]^−^	591.2028	−9.28	Osmanthuside B	163.0398, 145.0289	SDH
P79	16.62	C_41_H_62_O_15_	[M+HCOO]^−^	839.4047	−2.78	Betavulgaroside IV	631.3808, 455.3494	NX
P80	16.70	C_20_H_20_O_6_	[M−H]^−^	355.1177	−2.86	Coniferyl ferulate	340.0911, 311.1244, 296.1029	DG
P81	16.71	C_54_H_84_O_25_	[M+HCOO]^−^	1177.5327	3.71	Achyranthoside D methyl ester	969.4736	NX
P82	16.87	C_42_H_66_O_14_	[M−H]^−^	793.4392	1.56	Zingibroside R1	631.3831, 157.0138	NX
P83	16.98	C_21_H_22_O_8_	[M+H]^+^	403.138	−1.74	Nobiletin	217.0907	NX
P84	17.37	C_20_H_28_O_12_	[M+HCOO]^−^	505.1568	1.02	Apiopaeonoside	455.3560, 255.2385	NX
P85	17.61	C_30_H_48_O_6_	[M−H]^−^	503.3351	−5.31	23‐Hydroxytormentic acid	485.3254	NX
P86	17.79	C_16_H_35_NO_2_	[M+H]^+^	274.2736	−1.82	Lauryldiethanolamine	256.2619, 106.0853	DG
P87	18.00	C_16_H_22_O_4_	[M+H]^+^	279.1582	−3.00	Dibutyl phthalate	233.1524, 205.1583, 191.1059	DG, NX
P88	18.28	C_47_H_74_O_18_	[M−H]^−^	925.4793	−1.03	Chikusetsusaponin IV	793.4278	NX
P89	19.36	C_49_H_76_O_20_	[M−H]^−^	983.4844	−1.32	Achyranthoside C dimethyl ester	175.0242, 113.0235	NX
P90	19.53	C_12_H_12_O_2_	[M+H]^+^	189.0906	−2.49	9‐Butylidenephthalide	171.0804, 161.0595	DG
P91	19.88	C_48_H_76_O_18_	[M+H]^+^	941.5094	−1.11	Momordin IIa	777.4445, 615.3846, 455.3514	NX
P92	20.05	C_48_H_72_O_20_	[M+HCOO]^−^	1013.4694	9.42	Achyranthoside A	455.3530	NX
P93	20.06	C_41_H_60_O_15_	[M+HCOO]^−^	837.3902	−1.48	Achyranthoside iv	613.3710, 455.3530	NX
P94	20.08	C_42_H_66_O_14_	[M−H]^−^	793.4386	0.73	Chikusetsusaponin IVa	455.3530	NX
P95	20.22	C_16_H_24_O_10_	[M+HCOO]^−^	421.1312	−9.43	8‐Epiloganic acid	195.0640, 169.0867, 151.0766	SDH
P96	20.24	C_12_H_12_O_2_	[M+H]^+^	189.0907	−1.97	3‐Butylidenephthalide	143.0858	DG
P97	20.27	C_47_H_74_O_18_	[M−H]^−^	925.4835	3.48	Chikusetsusaponin Ib	763.4280, 455.3480	NX
P98	20.54	C_24_H_28_O_4_	[M+H]^+^	381.2051	−2.53	Levistilide A	191.106	DG
P99	20.63	C_18_H_30_O_2_	[M+H]^+^	279.2309	−3.55	Linolenic acid	277.2146, 141.0918	NX
P100	20.74	C_37_H_60_O_9_	[M+HCOO]^−^	693.4152	−9.71	Oleanolic acid 3‐O‐*β*‐D‐glucopyranoside‐6‐O‐methyl ester	113.0245	NX
P101	20.83	C_41_H_64_O_13_	[M−H]^−^	763.4244	−3.92	28‐Desglucosylchikusetsusaponin IV	613.3768	NX
P102	21.04	C_18_H_34_O_4_	[M−H]^−^	313.2370	−4.64	9,10‐Dihydroxy‐12Z‐octadecenoic acid	171.1030	DG
P103	21.20	C_12_H_14_O_3_	[M+H]^+^	207.1008	−3.62	4‐Hydroxy3‐butylphthalide	189.0903, 179.1056, 165.0906, 161.0958	DG
P104	21.31	C_42_H_66_O_13_	[M−H]^−^	777.4450	2.45	3‐O‐[*α*‐L‐rhamnopyranosyl‐ (1→3)‐*β*‐D‐glucurono‐ pyranosyl]oleanolic acid	777.4453, 631.3836, 455.3505	NX
P105	21.42	C_21_H_20_O_11_	[M+HCOO]^−^	493.0976	−2.27	Luteolin‐7‐O‐glucoside	387.0760, 357.0607, 327.0459, 285.0494	DG
P106	21.53	C_12_H_16_O_3_	[M+H]^+^	209.1168	−2.17	Senkyunolide K	173.0957, 163.1115, 145.1011, 117.0700	DG
P107	21.55	C_12_H_14_O_2_	[M+H]^+^	191.1059	−3.71	Ligustilide	173.0957, 163.1115, 155.0854,145.1011, 117.0700	DG
P108	21.55	C_24_H_28_O_4_	[M+H]^+^	381.2052	−2.22	Tokinolide B	191.1059, 173.0957, 163.1115, 155.0854, 145.1011	DG
P109	21.86	C_36_H_56_O_9_	[M−H]^−^	631.3844	−1.12	28‐Desglucosylchikusetsusaponin IVa	613.3746, 455.3484	NX
P110	21.93	C_10_H_10_O_4_	[M−H]^−^	193.0495	−5.97	Isoferulic acid	149.0592, 134.0366	DG, NX
P111	22.34	C_16_H_30_O_10_	[M+HCOO]^−-^	427.1814	−1.70	Shimaurinoside B	249.1336	SDH
P112	22.39	C_40_H_60_O_14_	[M−H]^−^	763.3851	−7.81	28‐Deglucosyl‐achyranthoside E	631.3840, 455.3504	NX
P113	22.41	C_36_H_58_O_8_	[M+HCOO]^−^	663.4119	0.78	Oleanolic acid 3‐O‐*β*‐D‐glucopyranoside	617.3952, 455.3504	NX
P114	22.50	C_17_H_34_O_2_	[M+HCOO]^−^	315.2535	−1.88	Margaric acid	116.9266	NX, SDH
P115	22.52	C_30_H_46_O_5_	[M−H]^−^	485.3258	−2.95	Quillaic acid	441.3349, 365.2832	NX
P116	23.79	C_18_H_32_O_3_	[M−H]^−^	295.2269	−3.16	13‐Hydroxy‐9,11‐octadecadienoic acid	195.1391	DG
P117	24.06	C_27_H_44_O_7_	[M−H]^−^	479.3060	9.45	Inokosterone	319.1927, 159.0990	NX
P118	24.26	C_48_H_76_O_19_	[M+H]^+^	957.5029	−2.6	*β*‐D‐glucopyranosyl 3*β*‐[O‐ *α*‐L‐rhamnopyranosyl‐(1‐ 3)‐O‐*β*‐D‐glucopyranuro‐ nosyloxy]oleanolate	613.5351	NX
P119	24.46	C_18_H_30_O_3_	[M−H]^−^	293.2096	−8.83	13‐Keto‐9Z,11E‐octadecadienoic acid	293.2083	NX
P120	25.32	C_12_H_14_O_2_	[M+H]^+^	191.1060	−3.41	3‐n‐Butylphthalide	173.0956	DG
P121	27.61	C_30_H_48_O_3_	[M−H]^−^	455.3532	0.34	Oleanolic acid	391.2917	NX
P122	29.39	C_30_H_46_O_6_	[M−H]^−^	501.3221	−0.10	26‐Hydroxyporicoic acid G	465.3035, 439.3256	NX
P123	29.82	C_18_H_32_O_2_	[M−H]^−^	279.2315	−5.38	Linoleic acid	261.2237	NX, DG,SDH
P124	31.27	C_36_H_58_O_9_	[M−H]^−^	633.3995	−2.04	Hederagenin‐28‐O‐*β*‐D‐glucopyranosyl ester	471.3449	NX
P125	31.35	C_21_H_30_O_4_	[M+HCOO]^−^	391.2150	6.07	Cortexolone	345.2001, 277.2108, 259.2133	NX
P126	31.68	C18H37NO	[M+H]^+^	284.2937	−3.9	Octadecanamide	102.0892	DG
P127	41.39	C_47_H_74_O_18_	[M+HCOO]^−^	971.4932	7.67	Pseudoginsenoside RT1	925.4782, 793.4392	NX

### 3.3. Identification of Major Types of Compounds and Analysis of Cleavage Patterns

#### 3.3.1. Terpenes

A total of 38 terpenoids were detected, mainly from achyranthis bidentatae radix and to a lesser extent from rehmanniae radix praeparata. Terpenoids are compounds and their derivatives with molecular formulae based on multiples of isoprene, and terpenoids can be categorised into different types based on the number of isoprenoids, such as monoterpenes, sesquiterpenes, diterpenes, dibenzoterpenes and so on. [42]. In the present study, 29 triterpenoids, seven cyclic enol ether terpene glycosides and one monoterpene were hypothesised, and the triterpenoids comprised 26 triterpene saponins and three free triterpenoids. Triterpenoid saponins, for example, have a wide range of biological activities, including anti‐inflammatory, anticancer and neuroprotective effects, usually in the C_3_ position and C_28_ position there are substitutions in the bisaccharide chain of glycosides, glycosides C_28_ carboxyl group is usually with the glucose group of the end group of the carbon into the ester, compared to the ether of the C position of the ether bond is more likely to be broken, so that the C_28_ position of the sugar chain is called *α*‐chain, the C_3_ position of the sugar chain is called the chain of the B. The C_2_ glycosidic group is partially susceptible to degradation under mass spectrometry collisions. Portion is vulnerable to shedding and loss under mass spectrometric collisions (five‐carbon sugars > six‐carbon sugars), and the glycosidic portion is susceptible to the loss of a range of neutral groups such as H_2_O, CO_2_ and HCOOH_37_.

**Figure 3:**
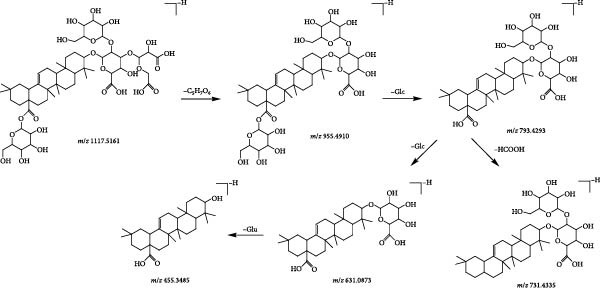
Possible fragmentation pathways of achyranthoside D.

Compound 72 (tR = 14.31 min) was used as an example to analyse the cleavage pattern of triterpenoid saponins. It produces a quasimolecular ion peak *m*/*z* 1117.5161 in the negative ion mode, and [M−H]^−^ in the secondary mass spectrum takes off −C_5_H_7_O_6_ to obtain the fragment ion of [M−H−C_5_H_7_O_6_]^−^ (*m*/*z* 955.4910), followed by the removal of 1 molecule of glucose to obtain the fragment [M−H−C5H7O6−Glc]^−^ (*m*/*z* 793.4293) ion; if −HCOOH is removed on this basis, the fragment ion of [M−H−C_5_H_7_O_6_−Glc−HCOOH]^−^ (*m*/*z* 731.4335) is formed, and if one molecule of glucose is removed directly, followed by one molecule of glucuronic acid (GluA) is removed to obtain the fragment ions of [M−H−C_5_H_7_O_6_−2Glc]^−^ (*m*/*z* 631.0873), [M−H−C5H7O6−2Glc−GluA]^−^ (*m*/*z* 455.3485) fragment ions. With reference to its cleavage fragmentation information, compound 72 was deduced to be Achyranthoside D. The cleavage diagram is shown in Figure 3.

**Figure 4:**
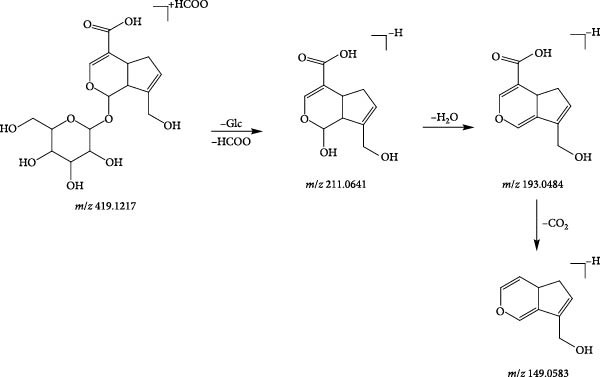
Possible fragmentation pathways of geniposidic acid.

Compound 22 (tR = 1.92 min) was used as an example to analyse the cleavage pattern of cyclic enol ether terpene glycosides. It produces a formate addition ion peak *m*/*z* 419.1217 in the negative ion mode, and [M−H]^−^ in the secondary mass spectrum takes off one molecule of glucose to form the fragment ion of [M−H−Glc]^−^ (*m*/*z* 211.0641); followed by the loss of one molecule of H_2_O to get the fragment ion of [M−H−Glc−H_2_O]^−^ (*m*/*z* 193.0484); if the one molecule of CO_2_ is removed, the fragment ion of [M−H−Glc−H_2_O−CO_2_]^−^ (*m*/*z* 149.0583) is formed. With reference to its cleavage fragmentation information, compound 22 was deduced to be geniposidic acid, and the cleavage pattern is shown in Figure 4.

Compound 121 (tR = 27.61 min) was used as an example to analyse the cleavage pattern of free triterpenoids. Its quasi‐molecular ion peak, *m*/*z* 455.3560, was produced in negative ion mode. The loss of one molecule of H_2_O by [M−H]^−^ in secondary mass spectrum gave the fragment ion of [M−H–H_2_O]^−^ (*m*/*z* 437.2693), followed by the loss of one molecule of CO to get the fragment of [M−H–H_2_O−CO]^−^ (*m*/*z* 409.2296); if the secondary mass spectrum was directly removed from the −HCOOH also gives the fragment ion of [M−H−HCOOH]^−^ (*m*/*z* 409.2296), and one molecule of H_2_O is lost to form the fragment ion of [M−H−HCOOH−H_2_O]^−^ (*m*/*z* 391.2220). With reference to the mass spectral information (Figure 5A) and the cleavage pattern (Figure 5C) with the oleanolic acid (Figure 5B), compound 121 was identified as oleanolic acid, and the mass spectra and cleavage diagram are shown in Figure 5.

Figure 5Mass spectra of oleanolic acid (sample (A) and standard (B)) and possible fragmentation pathways (C).

**Figure:**
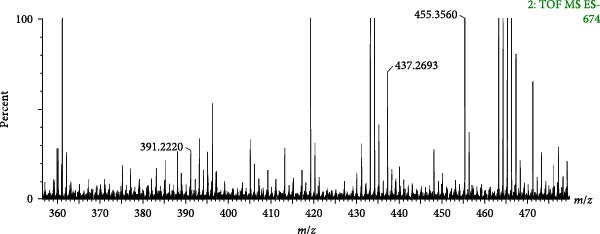
(A)

**Figure:**
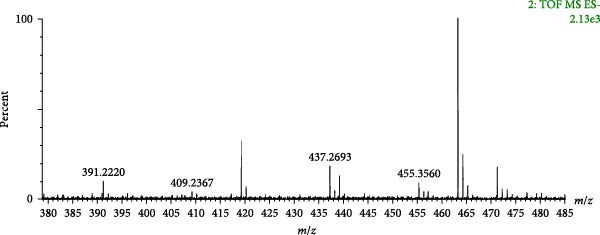
(B)

**Figure:**
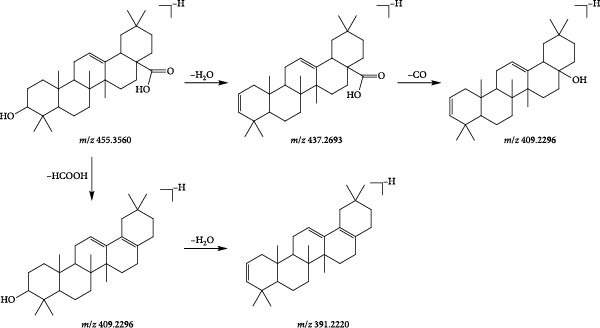
(C)

#### 3.3.2. Organic Acids

A total of 28 organic acids were identified based on mass spectrometry data. The herbs involved were achyranthis bidentatae radix, angelicae sinensis radix and rehmanniae radix praeparata. Organic acids are mainly composed of fatty acids, phenolic hydroxyl‐substituted aromatic rings and so on. The carbonyl groups are susceptible to cleavage under mass spectrometry conditions, resulting in the formation of fragment ions, or neutral loss of neutral groups such as H_2_O, COOH, CO_2_ and so on. A total of 13 phenolic acid components and 14 fatty acid components were identified by analysis. Compound 26 (tR = 2.26 min) was analysed as a representative of the phenolic acid components. Its quasi‐molecular ion peak *m/z* 193.0484 in the negative ion mode, and the neutral loss of one molecule of CO_2_ by [M−H]^−^ under the secondary mass spectrum, formed the molecular ion of [M−H−CO_2_]^−^ (*m*/*z* 149.0583); the continued detachment of −CH_2_ resulted in the formation of the molecular ion of [M−H−CO_2_‐− CH_2_]^−^ (*m*/*z* 135.0442) fragment ion; if −CH_2_ is removed directly under the secondary mass spectrum, [M−H−CH_2_]^−^ (*m*/*z* 179.0533) fragment ion is formed. Referring to the mass spectral information (Figure 6A) and the cleavage pattern (Figure 6C) with the ferulic acid control (Figure 6B), compound 26 was identified as ferulic acid.

Figure 6Mass spectra of ferulic acid (sample (A) and standard (B)) and possible fragmentation pathways (C).

**Figure:**
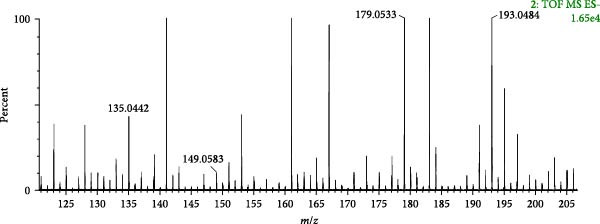
(A)

**Figure:**
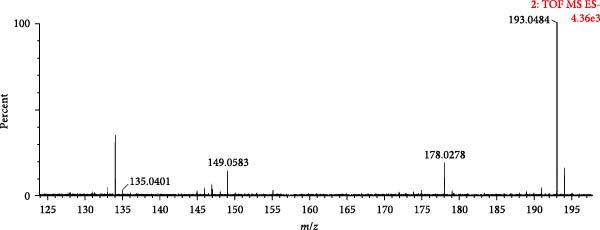
(B)

**Figure:**
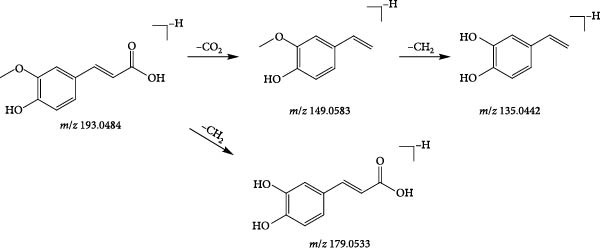
(C)

#### 3.3.3. Phthalides

**Figure 7:**
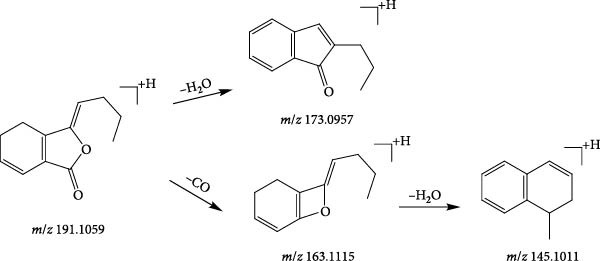
Possible fragmentation pathways of ligustilide.

A total of 11 phthalides were identified based on mass spectrometry data, all of which were derived from Angelicae Sinensis Radix. Phthalides are the main active components of Angelicae Sinensis Radix that exert the action of activating blood circulation and removing blood stasis. They can be classified into two major groups, that is, simple phthalides, dimerised phthalides, which usually lose H_2_O under the second level of mass spectrometry, CO, C_2_H_4_, C_3_H_6_, C_4_H_8_ and other molecules under secondary mass spectrometry to produce fragment ions [43]. Taking compound 107 (tR = 21.55 min) as an example, the quasimolecular ion peak [M+H]^+^ *m*/*z* 191.1059 in the positive ion mode, and the neutral loss of one molecule of water under the secondary mass spectrometry of [M+H]^+^ to form [M+H−H_2_O]^+^ (*m*/*z* 183.0957); if secondary, the direct loss of one molecule of CO in the mass spectrum gives the fragment ion [M+H−CO]^+^ (*m*/*z* 163.1115), and the neutral loss of one molecule of water continues to form the fragment [M+H−CO−H_2_O]^+^ (*m*/*z* 183.0957). The fragmentation information and the cleavage pattern were consistent with the literature [44], identifying compound 107 as ligustilide, and the cleavage pattern is shown in Figure 7.

#### 3.3.4. Phenylethanol Glycosides

**Figure 8:**
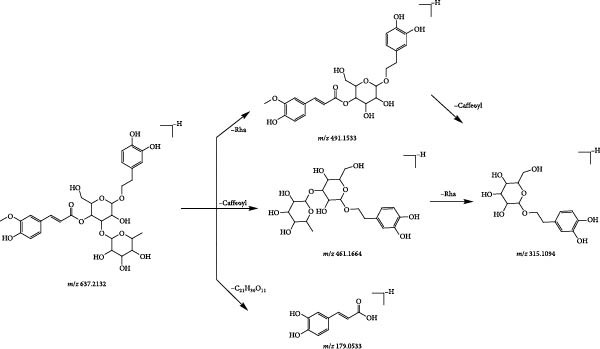
Possible fragmentation pathways of leucosceptoside A.

A total of nine phenylethanol glycosides were identified based on mass spectrometry data, all of which were derived from Rehmanniae Radix Praeparata. Phenylethanol glycosides are one of the main types of chemical constituents. This class of compounds undergoes mainly ester glycosidic or/and glycosidic bond breakage in the negative ionic mode, the former of which mainly loses caffeoyl (C_9_H_6_O_3_, 162) or acetyl groups neutrally (C_2_H_2_O, 42); the latter is prone to neutral loss of glucose residues (C_6_H_10_O_5_, 162) or rhamnose residues (C_6_H_10_O_4_, 146). Compound 37 (tR = 3.17 min) was used as an example to analyse the cleavage pattern of this class of compounds. Its quasimolecular ion peak [M−H]^−^ *m*/*z* 637.2132 in negative ion mode, such as secondary mass spectrometry [M−H]^−^, removes rhamnose, then gets [M−H−Rha]^−^ (*m*/*z* 491.1533) fragment ion; if secondary mass spectrometry is directly continued to remove caffeoyl group and then remove rhamnose, the formation of [M−H−Caffeoyl]^−^ (*m*/*z* 461.1664), [M−H−Rha]^−^ (*m*/*z* 491.1634), [M−H−Caffeoyl]^−^ (*m*/*z* 491.1634) and [M−H−Caffeoyl]^−^ (*m*/*z* 491.1634), respectively. 461.1664), [M−H−Caffeoyl‐Rha]^−^ (*m*/*z* 315.1094) fragment ions, respectively; if [M−H]^−^ was directly removed from −C_21_H_30_O_11_ under secondary mass spectrometry, the caffeic acid fragment [M−H−C_21_H_30_O_11_O]^−^ (*m*/*z* 179.0533) was obtained, and the combination of the fragmentation information and cleavage pattern (Figure 8). Compound 37 was hypothesised to be Leucosceptoside A.

#### 3.3.5. Phenylpropanoids

A total of 8 phenylpropanoids were identified based on mass spectrometry data, most of which were derived from Angelicae Sinensis Radix. In contrast, a few were derived from Rehmanniae Radix Praeparata, Achyranthis Bidentatae Radix. Phenylpropanoids are a class of natural compounds consisting of a benzene ring attached to three straight‐chain carbons, and are prone to lose neutral groups such as CH_3_ and CO_2_ in the secondary mass spectra [45]. The eight phenylpropanoids identified in this study can be classified as simple phenylpropanoids, coumarins and lignans based on structural differences. Compound 6 (tR = 1.22 min) was used as an example to analyse the cleavage pattern of this class of compounds. Its quasimolecular ion peak m/z 353.0871 was visible in the negative ion mode, such as [M−H]^−^ under the secondary mass spectrum stripped off −C_9_H_6_O_3_ and neutrally lost 1 molecule of H_2_O, forming fragment ions of [M−H−C_9_H_6_O_3_]^−^ (*m*/*z* 191.0530), [M−H−C_9_H_6_O_3_−H_2_O]^−^ (*m*/*z* 173.0419), respectively; such as [M−H]^−^ stripped of −C_7_H_12_O_5_ under secondary mass spectrometry, fragmentation of [M−H−C_7_H_12_O_5_]^−^ (*m*/*z* 179.0533) is formed and continued neutral loss of one molecule of H_2_O or one molecule of CO_2_ results in the formation of fragmentation of [M−H−H_2_O]^−^ (*m*/*z* 161.0454) or [M−H−CO_2_]^−^ (*m*/*z* 135.0442) ions. Combining the mass spectral information (Figure 9A) and the cleavage pattern (Figure 9C) with the chlorogenic acid (Figure 9B), compound 6 was confirmed to be chlorogenic acid.

Figure 9Mass spectra of chlorogenic acid (sample (A) and standard (B)) and possible fragmentation pathways (C).

**Figure:**
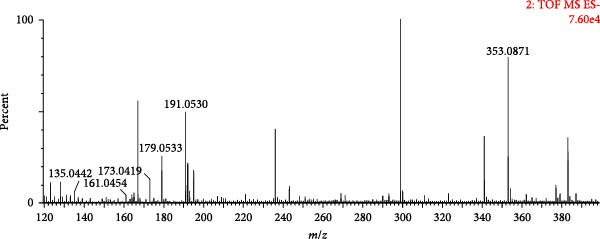
(A)

**Figure:**
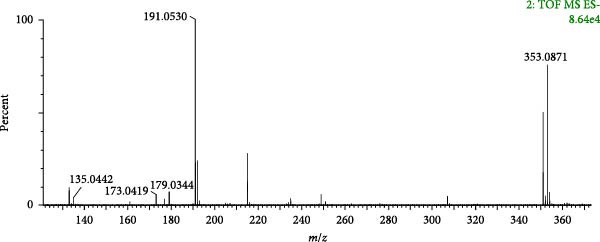
(B)

**Figure:**
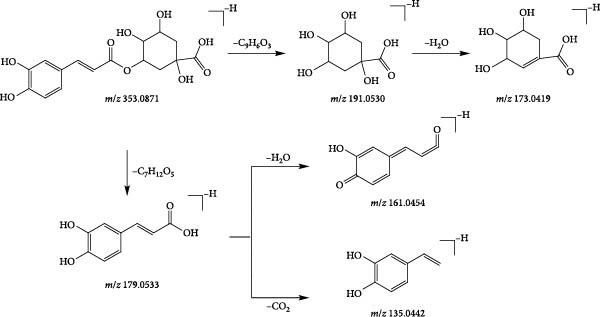
(C)

#### 3.3.6. Flavonoids

**Figure 10:**
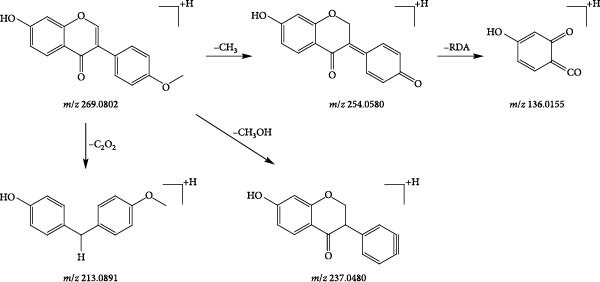
Possible cleavage pathways of formononetin.

A total of eight flavonoid compounds were identified, with the majority originating from achyranthis bidentatae radix and a few from angelicae sinensis radix. Flavonoids are widely distributed in nature, with their basic skeleton being 2‐phenylchromen‐4‐one, consisting of two benzene rings (A and B) connected by a three‐carbon chain. Compound 49 (tR = 5.73 min) exhibited a quasi‐molecular ion peak at *m/z* 269.0802 in positive ion mode. In the secondary mass spectrum, the [M+H]^+^ ion underwent a neutral loss of one molecule of CH_3_, forming [M+H−CH_3_]^+^ (*m*/*z* 254.0580). This was followed by further fragmentation via RDA cleavage, resulting in [M+H−C_8_H_6_O]^+^ (*m*/*z* 136.0155). Alternatively, the [M+H]^+^ ion could directly lose −CH_3_OH or −C_2_O_2_, forming [M+H−CH_3_OH]^+^ (*m*/*z* 237.0480) or [M+H−C_2_O_2_]^+^ (*m*/*z* 213.0891), respectively. Based on the precise molecular mass and fragmentation patterns, compound 49 was tentatively identified as formononetin, with the fragmentation pattern illustrated in Figure 10.

**Figure 11:**
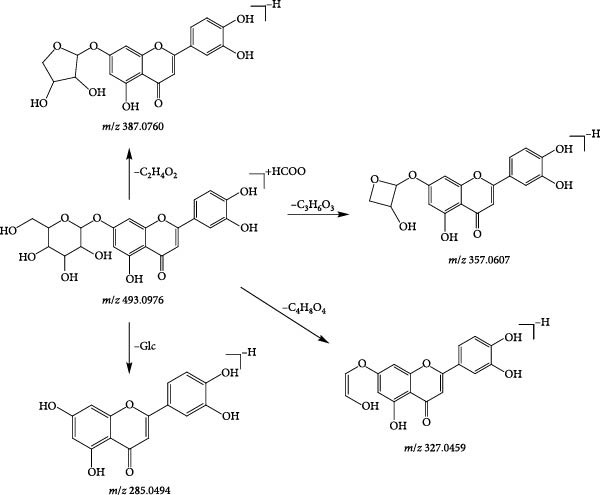
Possible cleavage pathways of luteolin‐7‐O‐glucosid.

A common cleavage pathway for isopentenyl‐free flavone oxyglycosides is the loss of the sugar group to produce a series of deglycosylated fragments, followed by the susceptibility of their glycosides to secondary bombardment. Compound 102 (tR = 21.42 min) was taken as an example to analyse the cleavage pattern of this class of compounds. Its formate addition ion peak m/z 493.0976 was visible in the negative ion mode, and the fragment ions of [M−H]^−^ stripped of −C_2_H_4_O_2_ to form [M−H−C_2_H_4_O_2_]^−^ (*m*/*z* 387.0760) in the secondary mass spectrum; if stripped of −C_3_H_6_O_3,_ −C_4_H_8_O_4_, then [M−H−C_3_H_4_O_3_]^−^ (*m*/*z* 357.0607) and [M−H−C_4_H_8_O_4_]^−^ (*m*/*z* 327.0459) fragment ions; if the breakage of the glucose group occurs directly under the secondary mass spectrum, [M−H−Glc]^−^ (*m*/*z* 285.0494) fragment ions are formed. Based on the exact molecular mass of the compounds and the cleavage pattern, compound 102 was identified as luteolin‐7‐O‐glucoside, and the cleavage diagram is shown in Figure 11.

#### 3.3.7. Steroids and Ketones

Steroids and ketones are two of the main compounds in achyranthis bidentatae radix. Their main structural features are the C_6_ ketone group, C_7_ double bond, and an oxygen‐containing functional group side chain consisting of 8–10 carbon atoms attached to C_17_ of the steroid parent nucleus, and a cis‐consistency of the A/B ring on the parent nucleus, with the B/C ring being trans. Most of the C/D ring is being trans. Under the negative ion mode, the MS spectra of these compounds have three series of peaks: (1) [M−nH_2_O−H]‐peak; (2) fragment peaks after C_17_−C_20_ cleavage and (3) fragment peaks after C_20_−C_22_ breakage. Based on the mass spectrometry data, a total of eight steroids and ketones were identified in this study, all of which were derived from achyranthis bidentatae radix. Compound 23 (tR = 1.93 min) was used as an example to analyse the cleavage pattern of this class of compounds. It produces a quasimolecular ion peak *m*/*z* 479.3041 in negative ion mode, such as [M−H]^−^ degradation of −C_8_H_16_O_3_ under the secondary mass spectrum to form the fragmentation ion of [M−H−C_8_H_16_O_3_]^−^ (*m*/*z* 319.1927), and continues to degradation of one molecule of H_2_O, it forms [M−H−C_8_H_16_O_3_−H_2_O]^−^ (*m*/*z* 301.0117) fragmentation; if [M−H]^−^ is directly stripped of −C_19_H_28_O_4_ under secondary mass spectrometry, the fragment ion [M−H−C_19_H28O_4_]^−^ (*m*/*z* 159.0990) is formed. Based on the exact molecular mass of the compound and the cleavage pattern, compound 23 was identified as ecdysterone.

Compound 39 (tR = 3.31 min), which produces a quasimolecular ion peak m/z 519.2971 in the negative ion mode, such as [M−H]^−^ deletion of −C_19_H_28_O_4_ to form the fragment ion of [M−H−C_19_H_28_O_4_]^−^ (*m*/*z* 199.0968) in the secondary mass spectrum, and the formation of [M−H−[M−H−C_10_H_16_O_4_]^−^ (*m*/*z* 319.1864), if the direct deletion of −C_10_H_16_O_4_ is continued. C_10_−H_16_O_4_]^−^ (*m/z* 319.1864), and continued neutral loss of one molecule of H_2_O results in the formation of the fragment ion [M−H−C_10_H_16_O_4_−H_2_O]^−^ (*m*/*z* 301.1767). Combining the mass spectral information (Figure 12A) and the cleavage pattern (Figure 12C) with the cyasterone (Figure 12B), compound 39 was confirmed to be cyasterone.

Figure 12Possible cleavage pathways of cyasterone.

**Figure:**
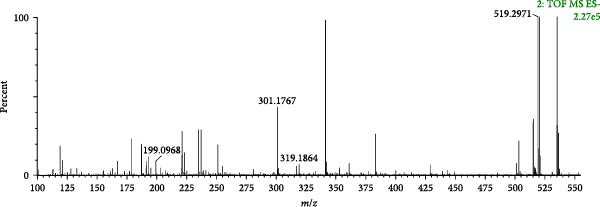
(A)

**Figure:**
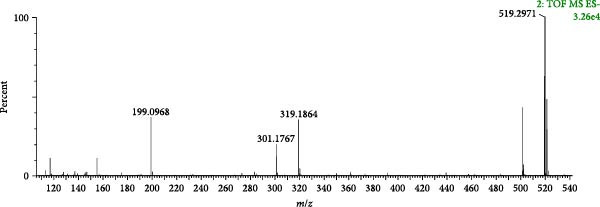
(B)

**Figure:**
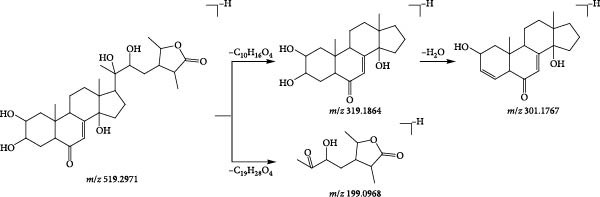
(C)

### 3.4. Identification of SND Toxic Components and Targeted Pharmacological Analyses

#### 3.4.1. Identification of Active Ingredients and Related Targets

According to the screening criteria of the active ingredients mentioned earlier, a total of 47 compliant active ingredients were identified in SND (Table S3). After searching, merging and de‐weighting in Swiss target prediction, SuperPred and SEA databases, a total of 1341 SND‐related targets were obtained.

#### 3.4.2. SND and NONFH‐Related Target Prediction

The GSE123568 dataset is a microarray analysis of expression profiles from peripheral blood, including 30 NONFH patient samples and 10 healthy control samples. The DEGs between NONFH samples and normal samples were screened with |logFC| > 0.5 and *p* < 0.05, and the results are presented as volcano plots (Figure 13A). A total of 1780 genes were identified in the analysed results, of which 988 genes were up‐regulated and 792 genes were down‐regulated.

Figure 13Weighted network analysis between NONFH and healthy samples. (A) Volcano plot of DEGs between NONFH and healthy samples. (B) Determination of soft threshold power. (C) Cluster tree dendrogram of co‐expressed modules. (D) Representation of clusters of module signature genes. (E) Correlation analysis of module signature genes with disease status. (F) MEblue module genes and NONFH clustering significant scatter plots among the differential genes.

**Figure:**
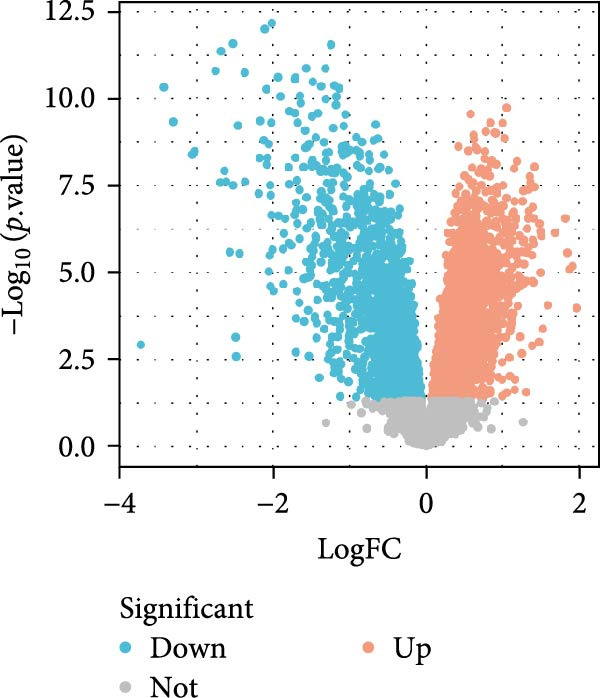
(A)

**Figure:**
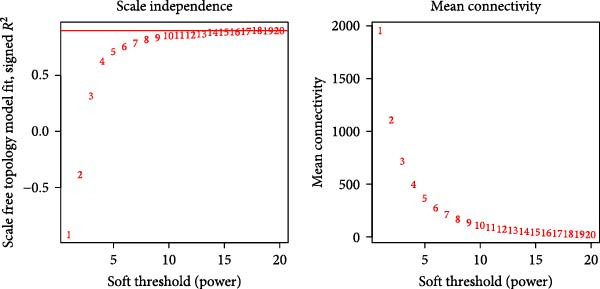
(B)

**Figure:**
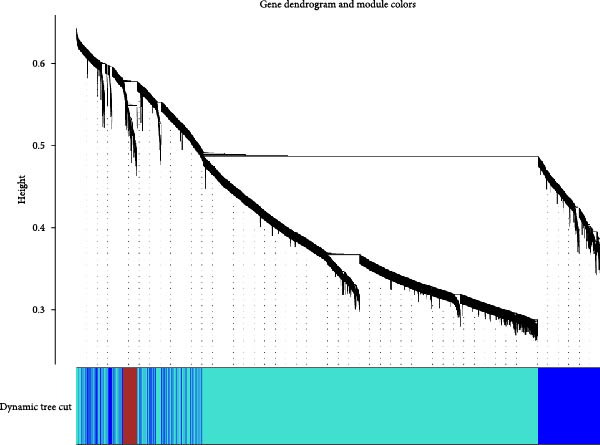
(C)

**Figure:**
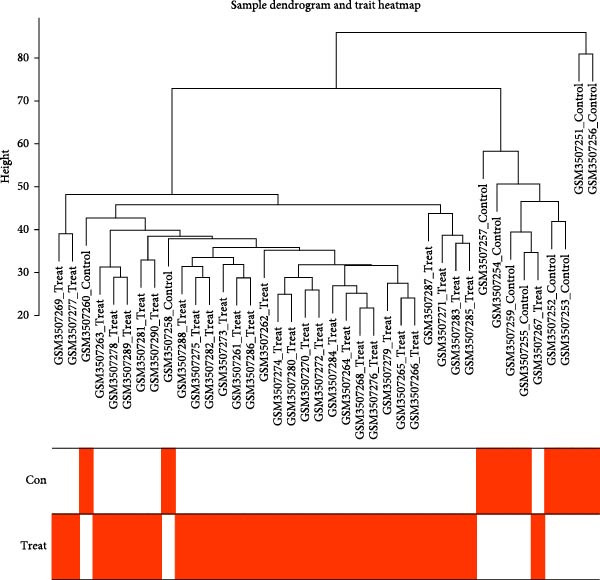
(D)

**Figure:**
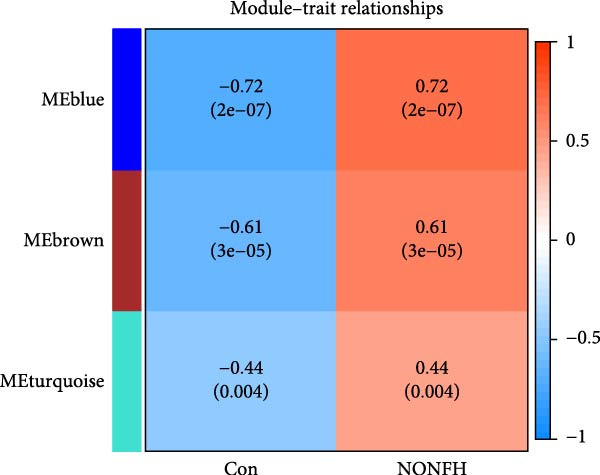
(E)

**Figure:**
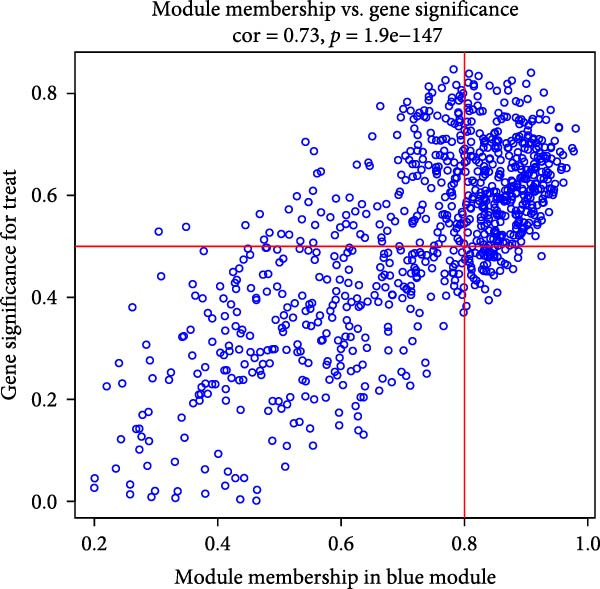
(F)

The WGCNA co‐expression network was constructed based on GSE123568, and a scale‐free network was constructed using *β* = 11 and *R* ^2^ = 0.9 as criteria (Figure 13B). 4709 genes were classified into 3 key modules with different colours, and the TOM matrices of the genes associated with all the modules are visualised in Figure 13B. Control‐NONFH group relationship analysis showed (Figure 13C) that the most significant module was MEblue (*n* = 439, *p* = 2e^−7^). Figure 13F shows the correlation of the above MEblue module with the NONFH group genes.

#### 3.4.3. Target Organ and GO/KEGG Enrichment Analysis

NONFH, SND‐related targets were combined to take intersections, and a total of 46 cross‐targets were identified (Figure 14A). These targets were considered as potential targets for SND for NONFH (Table S4). The enrichment of the 46 potential targets in various tissues and organs of the human body was explored using the DAVID tool. The results showed that four potential targets were significantly enriched in monocyte (*p* = 3.78e^−4^), 19 targets were enriched in the placenta (*p* = 0.001) and five targets were enriched in T‐cells (*p* = 0.005) (Figure 14B).

Figure 14Graphs of potential target identification and GO/KEGG with target organ enrichment analysis. (A) Venn diagram of potential target identification. (B) Enrichment map of potential target tissues and organs. (C) Double histogram of BP, MF and CC classes ranked in the top 10 of *p*‐value. (D) Pathways bubble diagram of the top 10 of *p*‐value.

**Figure:**
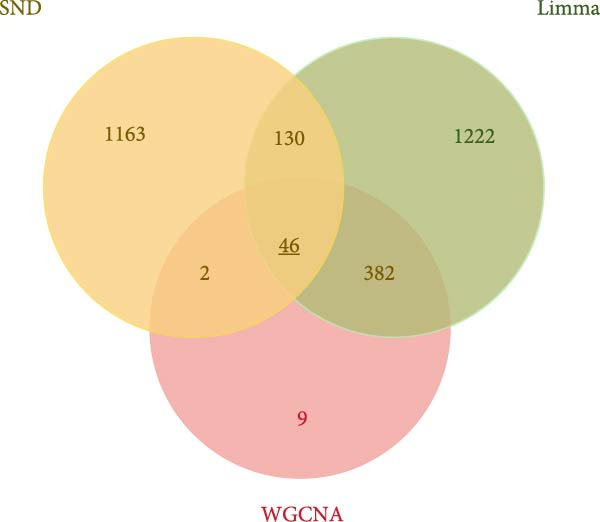
(A)

**Figure:**
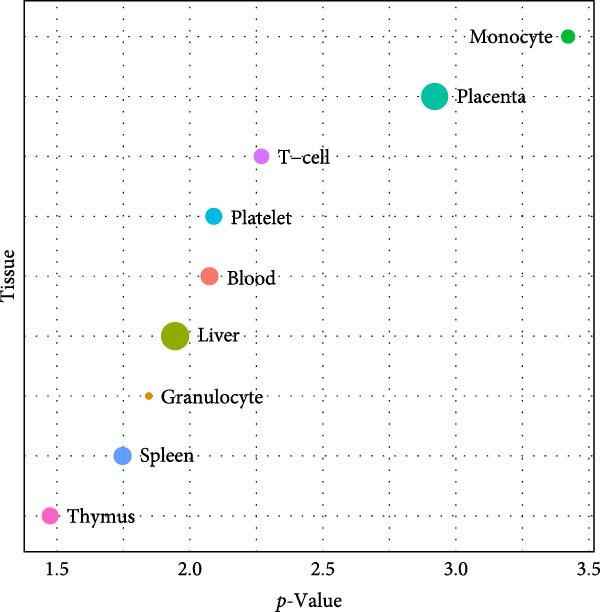
(B)

**Figure:**
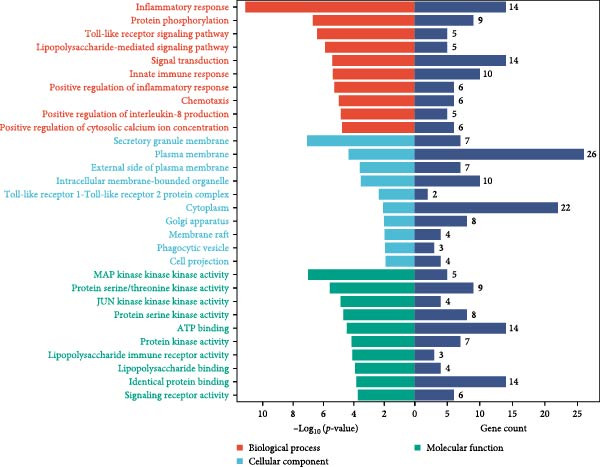
(C)

**Figure:**
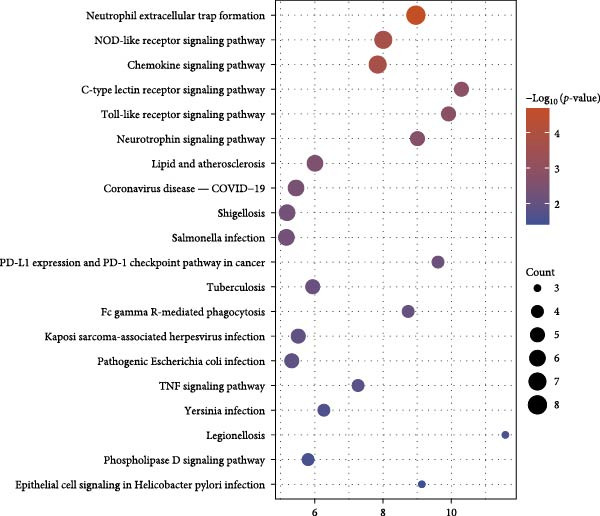
(D)

In addition, 46 targets were enriched in a total of 111 BP, 40 MF, 27 CC and 31 pathways (Table S5). *p*‐value was used as the screening criterion to visualise the top 10 BP, MF and CC items in the form of double histograms (Figure 14C), and the top 20 pathways in the form of bubble plots (Figure 14D). Among them, the pathway with the smallest *p*‐value was NETs, and others included hypoxia‐inducible factor‐1 (HIF‐1) signalling pathway, efferocytosis, neuroactive ligand–receptor interaction and so on.

#### 3.4.4. Identification of Candidate Targets Based on Multiple Machine Learning Models and Interpretable SHAP Methods

In order to further screen the candidate targets of SND for NONFH treatment, eight machine learning models were constructed based on 46 potential targets to accurately identify feature genes. The ‘DALEX’ package was executed to compare the above eight models and analyse the residual distribution of each model. The results showed that the residuals of SVM models were relatively low (Figure 15A). Subsequently, in order to evaluate the discriminative performance of the eight models, we plotted the ROC curves of the eight models based on 5‐fold cross‐validation (Figure 15B). The results show that the three models with the best discriminative performance are XGB (AUC = 0.852), SVM (AUC = 0.778) and RF (AUC = 0.741), in that order. Figure 15C illustrates the top 10 significant targets under root mean square error (RMSE) for the eight models (Figure 15C). Combining the above analyses, the SVM method was identified as the best model in this study, and the 10 targets associated with this model (Acid Phosphatase 1 (ACP1), NLRP3, ITM2B, haematopoietic cell kinase (HCK), IGF2R, C‐X‐C Chemokine Receptor 2 (CXCR2), C5AR1, NADH: ubiquinone oxidoreductase subunit A3 (NDUFAF3), FFAR2 and platelet‐activating factor receptor [PTAFR]) were used as the candidate targets for the subsequent study.

Figure 15Construction and SHAP evaluation of 8 machine models. (A) Residual box plots of the eight machine learning models, with RMSE indicated by red dots. (B) ROC curves of the 8 machine learning models based on fivefold cross‐validation of the test set. (C) Important functions of the 8 machine learning models. (D) Ranking of the 10 candidate targets in terms of their impact on the SVM model. (E) The left side of the *X*‐axis represents factors that lead to negative changes in prediction results, while the right side represents positive changes in factors; *Y*‐axis represents the importance of candidate targets on disease impact. (F) ROC validation of five key targets.

**Figure:**
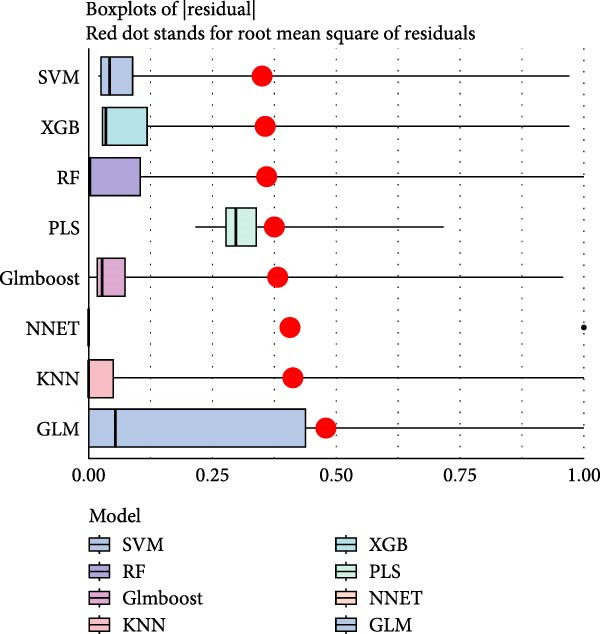
(A)

**Figure:**
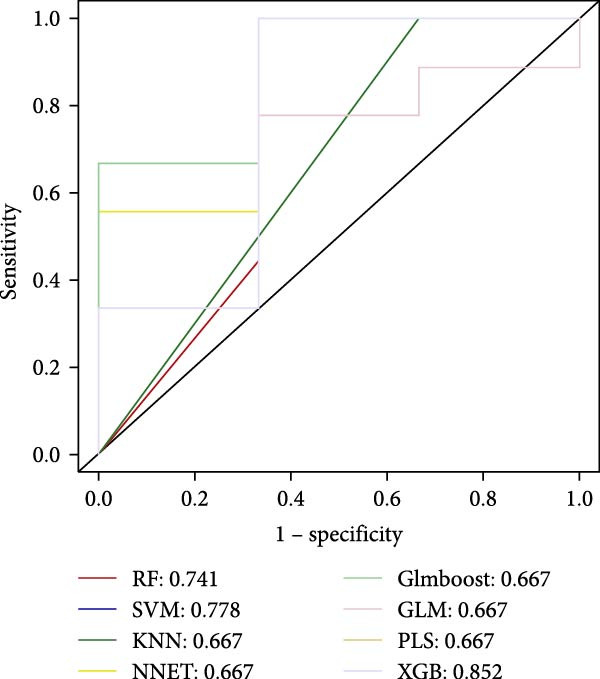
(B)

**Figure:**
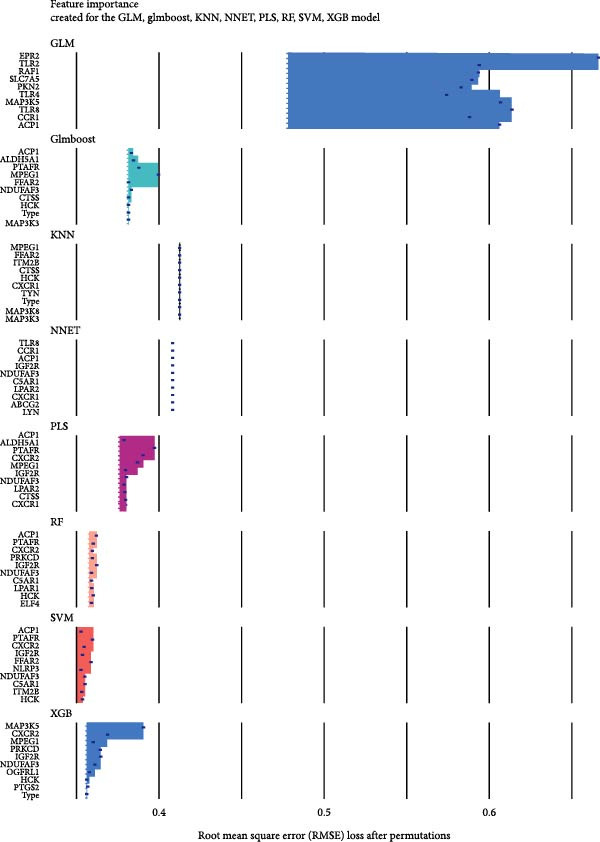
(C)

**Figure:**
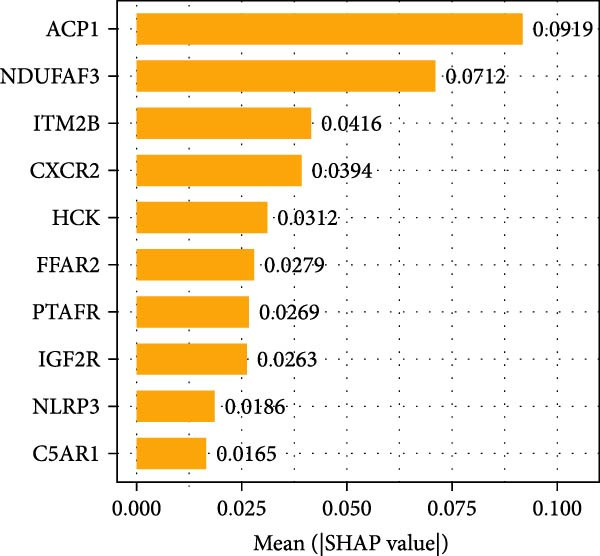
(D)

**Figure:**
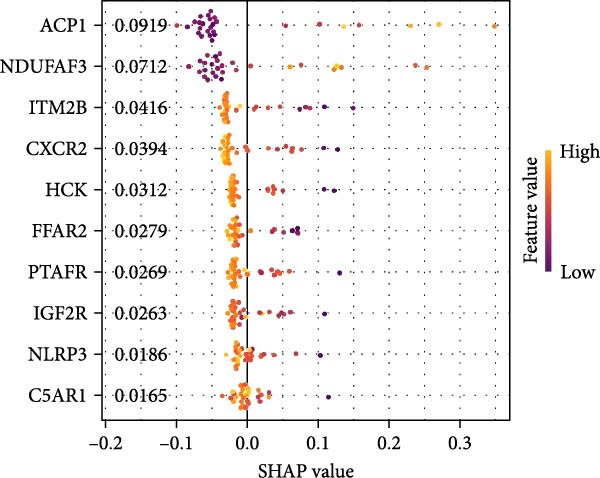
(E)

**Figure:**
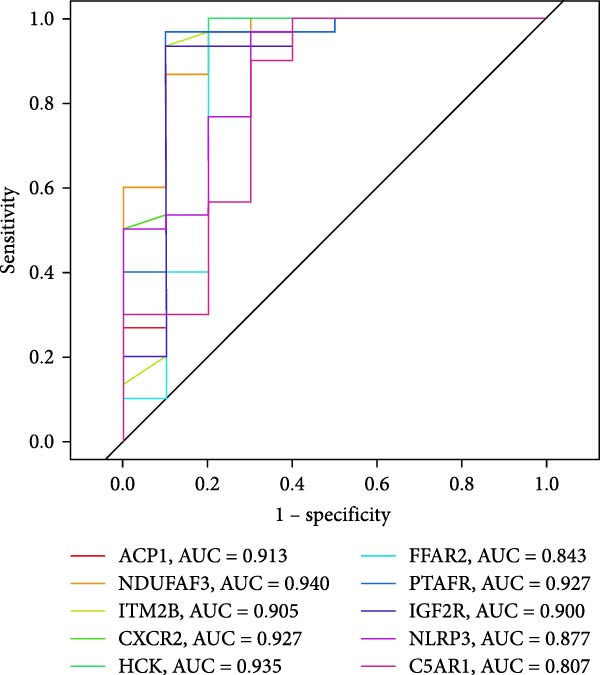
(F)

The SHAP method was used to analyse the influence of the above 10 candidate targets on the SVM model, to better predict and elucidate the mechanism of action of SND for the treatment of NONFH. Figure 15D demonstrates the influential ranking of the 10 targets. The influence of each target on the model output is shown in Figure 15E, which shows that the scatter on the right side of the X‐axis represents a positive correlation with NONFH, that is, a protective factor, whereas the scatter on the left side of the X‐axis represents a negative correlation, that is, a risk factor; in addition, yellow colour represents a high level of expression, and purple colour represents a low level of expression. For example, low expression of ACP1 increases the risk of NONFH, while low expression of HCK has a favourable effect on the disease. Finally, we validated the ROC curves of the 10 candidate targets (Figure 15F). Taken together, these analyses demonstrated that ACP1, HCK, CXCR2, NDUFAF3 and PTAFR had relatively high diagnostic value and a satisfactory predictive effect, and thus were regarded as key targets for SND in NONFH.

#### 3.4.5. NONFH Immune Infiltration Analysis

We performed immune infiltration analysis between NONFH and healthy samples (Figure 16A,B). The results showed that NONFH samples exhibited low‐level infiltration in B‐cell memory, dendritic cells and activated cells; notably, although there was no significant difference between NONFH and healthy samples in neutrophils cells, there was a higher expression abundance in both (Figure 16C), which shows that NONFH is correlated between the development and the immune system. Subsequently, we analysed the immune infiltration of the five key targets between NONFH and healthy samples (Figure 16D). The results showed that PTAFR, HCK and CXCR2 all had positive and strong correlations with neutrophils (*p*  < 0.001), and significant negative correlations with T cells, CD8 and eosinophils. Overall, there was a relatively close correlation between PTAFR, HCK, CXCR2 and a variety of immune cells, which may play a key role in the development and immune infiltration of NONFH.

Figure 16Analysis of immune infiltration between NONFH and healthy samples. (A) Relative abundance of NONFH and healthy samples among 22 infiltrating immune cells. (B) Correlation of 22 infiltrating immune cells. (C) Box plot of immune infiltrating cells between NONFH and healthy samples. (D) Correlation analysis between five key targets and immune cells.

**Figure:**
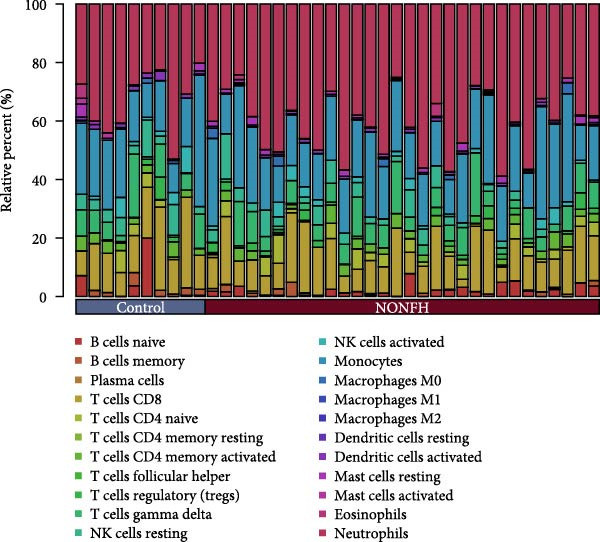
(A)

**Figure:**
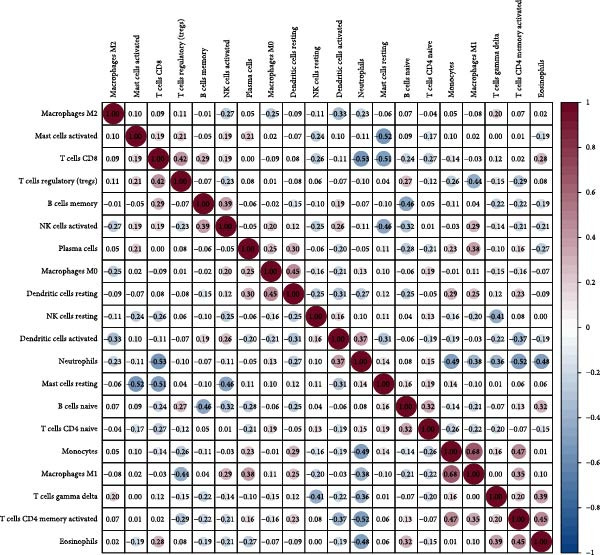
(B)

**Figure:**
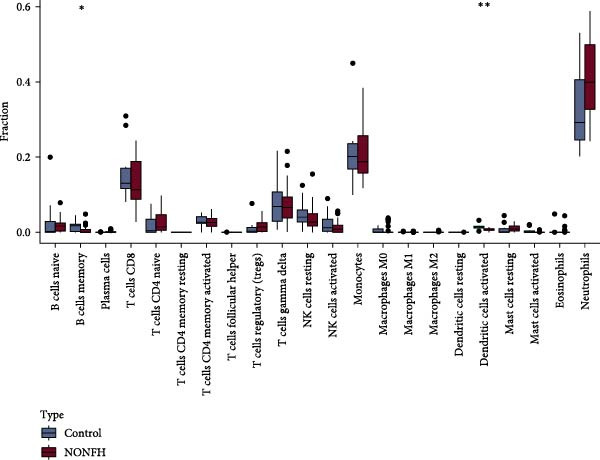
(C)

**Figure:**
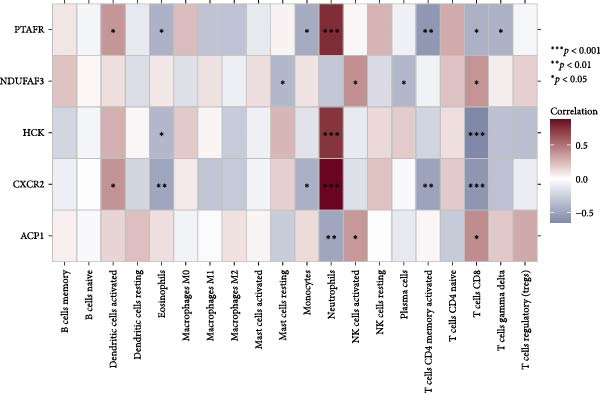
(D)

#### 3.4.6. Key Target Validation

Five key targets from the SVM model were selected, and their expression levels were analysed in NONFH and healthy samples. The results showed that the expression of ACP1 and NDUFAF3 was significantly lower in the NONFH group than in the control group. In contrast, the expression levels of HCK, CXCR2 and PTAFR were significantly higher (Figure 17A). We analysed the relationship between these targets and showed a significant positive correlation between PTAFR and CXCR2, HCK (OR: 0.79/0.64, *p* < 0.001); in addition, there was a significant negative correlation between ACP1 and CXCR2, HCK (OR: −0.68/−0.76, *p* < 0.001), as detailed in Figure 17B.

Figure 17Validation of key targets. (A) Differential expression of key targets in NONFH and control. (B) Correlation analysis between key targets.

**Figure:**
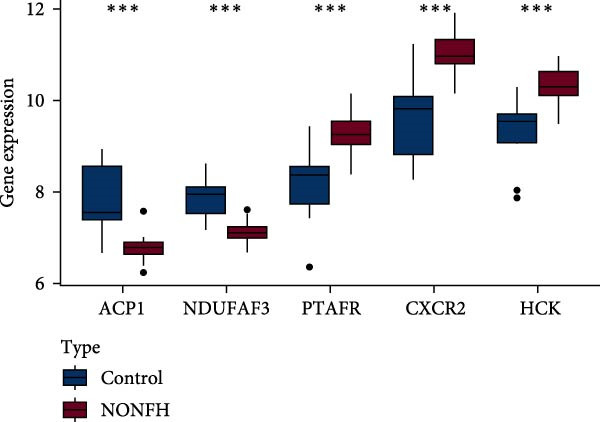
(A)

**Figure:**
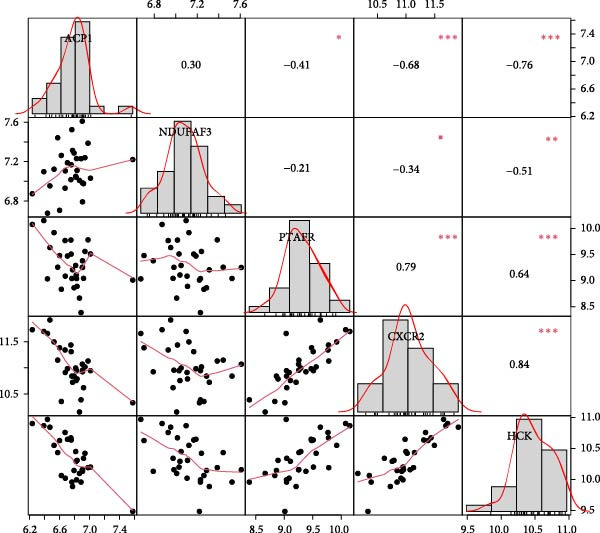
(B)

#### 3.4.7. MD and Kinetic Simulation Validation

MD was carried out using the five key targets as receptor macromolecules and their corresponding SND active ingredients as ligand macromolecules, and the specific docking information between the receptors and ligands is shown in Table S6. The smallest binding energy was found to be that of HCK‐Chrysophanic acid (−8.7 kcal/mol), followed by those of HCK‐Epinortrachelogenin (−8.3 kcal/mol), PTAFR‐Senkyunolide F (−7.8 kcal/mol), ACP1‐Quillaic acid (−7.5 kcal/mol), ACP1‐Kaempferol (−7.3 kcal/mol) and so on. We chose the binding energy <−7.5 kcal/mol to show the results (Figure 18). The docking results are shown (Figure 18A–D). It is evident that chrysophanic acid in SND may exert a therapeutic effect on NONFH by regulating HCK.

Figure 18Visualisation of MD and HCK‐chrysophanic acid MDS results. (A) Chrysophanic acid/Epinortrachelogenin‐HCK. (B) Senkyunolide F‐PTAFR. (C) Quillaic acid‐ACP1. (D) Free HCK backbone vs. backbone‐complex RMSD plots. (E) Free HCK vs. complex RMSF plots. (F) Complex hydrogen bonding diagram. (G) Free HCK vs. complex Rg diagram. (H) Complex FEL diagram and the best conformation in the energy low‐lying region.

**Figure:**
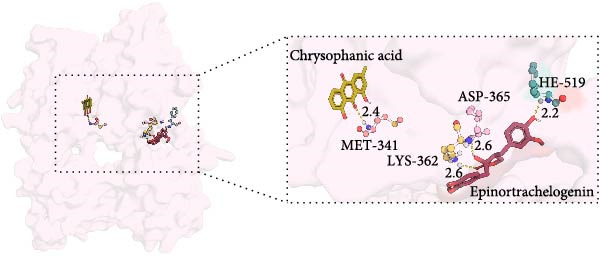
(A)

**Figure:**
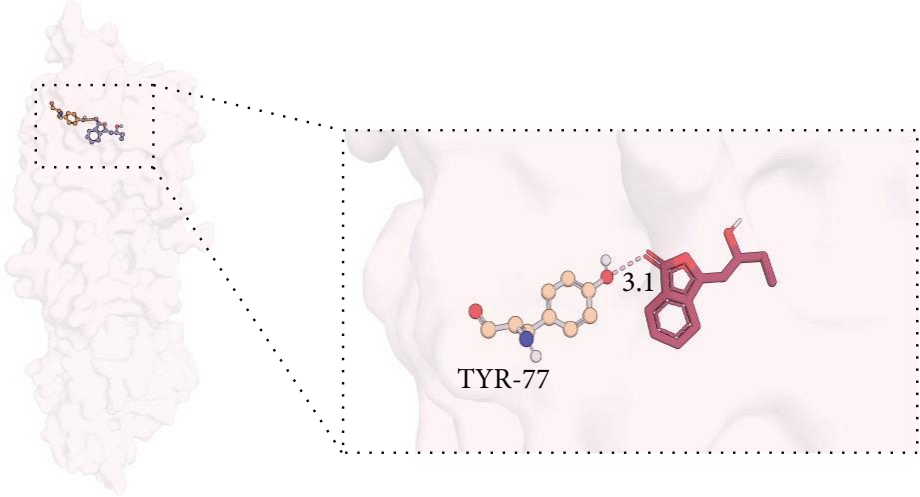
(B)

**Figure:**
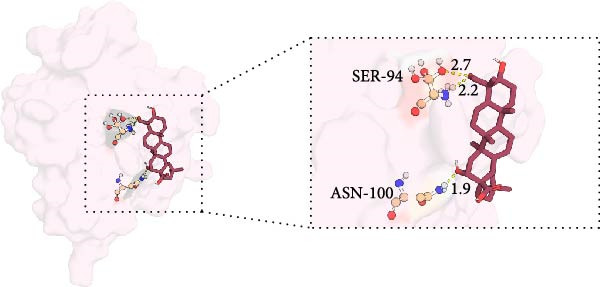
(C)

**Figure:**
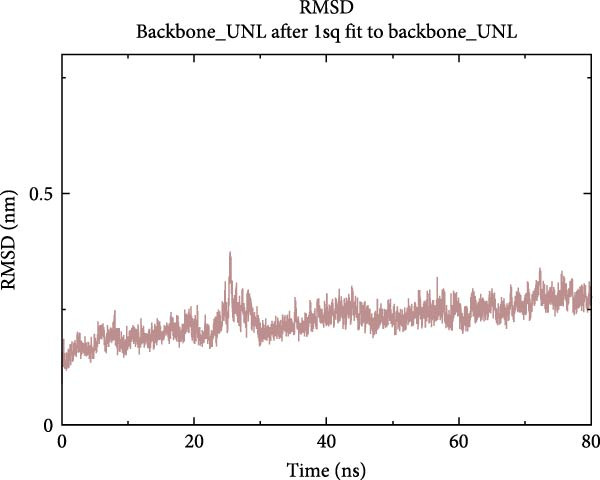
(D)

**Figure:**
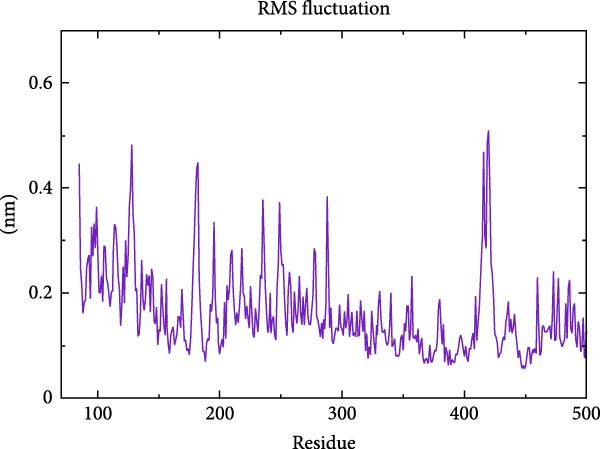
(E)

**Figure:**
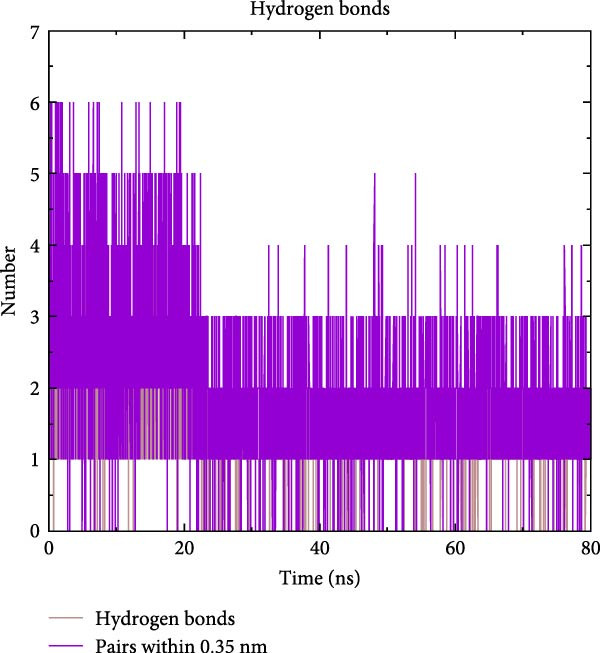
(F)

**Figure:**
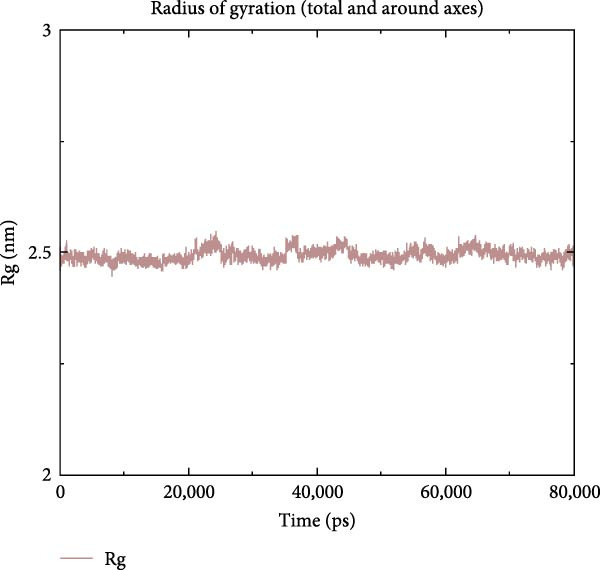
(G)

**Figure:**
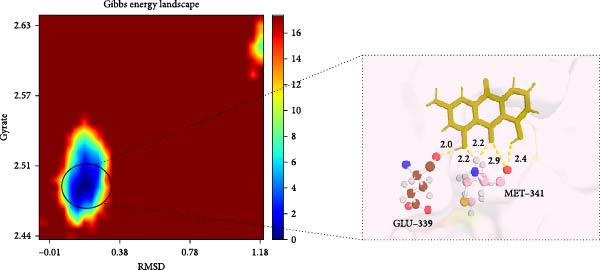
(H)

The above molecular docking results indicate that the binding affinity of the HCK‐chrysophanic acid complex is strong. We further used 80 ns MDS to analyse the stability and conformational changes of the complex. In general, the RMSD value is inversely proportional to the conformational stability; that is, the smaller the former, the higher the conformational stability. As shown in Figure 18D, the RMSD values of the free HCK backbone and HCK backbone–Resveratrol complex fluctuated in the early stage of the simulation (the first 40 ns), and then tended to be stable in the last 40 ns, indicating that the binding of the ligand to the HCK backbone would not have a large impact on the protein backbone itself, and that the absence of a break in the RMSD curve represents a strong binding ability of the two. RMSF plots showed changes in HCK amino acid residues, with most simulations causing minor fluctuations in the amino acid structure and very few showing minor structural changes (Figure 18E). The RMSF values of free HCK and HCK‐chrysophanic acid complexes were all ≤0.6 nm, with minor fluctuations but overall high stability. The number and density of hydrogen bonds in the complexes can reflect their binding strength to some extent. As shown in Figure 18F, the number and density of hydrogen bonds and the strength of HCK‐Chrysophanic acid complexes were high, and the maximum number of hydrogen bonds could reach 6, with strong binding stability. The results of the radius of rotation (Rg) analysis showed that the radius of rotation of free HCK and HCK‐chrysophanic acid complexes was relatively stable, indicating that the formation of the complexes would not affect the protein conformation on a large scale (Figure 18G). The FEL can reflect the change in the free energy of the substance during the simulation process, and the darker colour in the FEL represents the lower binding energy. At the same time, the calculation of the complexes can provide the basis for the subsequent extraction of the best characteristic conformation. In this study, two data, RMSD and Rg, were used to establish the FEL of the HCK‐Chrysophanic acid complex. As shown in Figure 18H, there exists a low‐energy region, that is, a relatively stable state during the simulation of the structure of the complex, and the conformations in the low‐energy region were extracted and visualised to show their docking state.

## 4. Discussion

NONFH is a chronic, destructive disease closely related to alcoholism, glucocorticoid medication and other factors, which involves progressive osteonecrosis and may lead to collapse of the femoral head and loss of hip function, which seriously affects patients’ quality of life [46, 47]. Although the pathophysiological mechanism of NONFH has not been elucidated yet, most scholars believe that the disease is closely related to abnormal lipid metabolism, vascular injury, inflammation, altered bone cell physiology, insufficient blood supply and oxidative stress [11, 48, 49]. TCM has a long history of treating bone diseases, and studies have shown that the use of TCM in treating patients with NONFH can effectively alleviate their pain and other symptoms, and at the same time improve their quality of life, with safe and effective results [50, 51]. The use of TCM in treating patients with NONFH has also been shown to be effective in improving the quality of life of patients with NONFH.

In this study, the literature on TCM treatment of NONFH was collected from the databases of CNKI, Wanfang and PubMed using TCMISS V2.5 software. Through this process, it was found that ‘SND’ was the drug pairing with the highest confidence level. *Achyranthes bidentata* Blume, a plant of the Amaranthaceae family, is a commonly used CHM for TCM. Current studies have shown that more than 270 active compounds have been identified in *A. bidentata* Blume, which can be classified as flavonoids, saponins, ecdysteroids, ketosteroids and so on, and have a wide range of pharmacological effects, such as inhibition of apoptosis of chondrocytes, modulation of bone and mineral metabolism, and other effects [51–53]. In the study of Yan et al. [54], it was found that AB50. A fructooligosaccharide from *A. bidentata* Blume could regulate bone metabolism‐related indexes, such as up‐regulating PINP and OCN, and down‐regulating TRAP and CTX; and increase the bone mineral density (BMD) of the femur in OVX rats, and so on, playing a role in stimulating bone formation [54]. In a study by Jiang et al. [52], *A. bidentata* extract increased BMD and promoted angiogenesis in steroid‐induced ONFH rats by improving the microarchitecture of cancellous bone; in addition, it inhibited osteoclast differentiation and activated bone formation markers, which acted as an osteoprotective agent in NONFH [52, 54]. Previous studies have demonstrated the importance of osteocyte physiology in the pathogenesis of NONFH, with osteoblasts, osteoclast apoptosis and abnormal osteoclast activity being among the leading causes of NONFH [48]. In our study, radix rehmanniae preparata was found to be a commonly used TCM, but no separate study of it on NONFH was found at this time. Radix rehmanniae preparata is a concocted and processed product of *Rehmannia glutinosa* Libosch (*Rehmannia glutinosa* Libosch), a plant of the Genusaceae family, which is a commonly used tonic herb in TCM. More than 100 active compounds have been identified, which can be classified as glycosides, polysaccharides, flavonoids, steroids and so on. They have a wide range of pharmacological effects, such as analgesic, sedative, anti‐inflammatory, antioxidant, anti‐tumour and immunomodulatory and are commonly used in the treatment of bone metabolism disorders, such as osteoporosis, osteoarthritis, and so forth [54–56]. Thus, we found that Radix Rehmanniae Preparata was able to promote bone formation by inhibiting the biosynthesis of steroid hormones and by regulating the expression levels of RUNX2 and OPN proteins through the perspective of the physiological role of osteoblasts [57].

In addition, in Luo et al.’s [58] study, Radix Rehmanniae Preparata was found to promote the increase of MiR‐29a–3p levels in serum‐derived exosomes, which in turn inhibited Nuclear Factor I/A to enhance bone formation. Therefore, we hypothesise that Radix Rehmanniae Preparata may synergise with anti‐osteonecrosis effects by promoting bone formation as well as antioxidant effects. Radix Angelicae Sinensis is the dried root of *Angelica sinensis* (Oliv.) Diels, a family of Umbelliferae, which has significant medicinal value as an important blood tonic in TCM. *A. sinensis* contains a variety of active constituents, mainly including volatile oils, flavonoids, polysaccharides and other components [59]. These constituents confer a variety of pharmacological effects such as antioxidant, anti‐inflammatory, regulation of lipid metabolism and promotion of blood circulation, especially in the promotion of osteogenesis and angiogenesis [59–61]. In Li et al.’s study, it was found that calycosin, the active ingredient in Radix Angelicae Sinensis, could alleviate bone loss through the MAPK signalling pathway and promote bone formation by regulating the expression of OPG, RANKL mRNA and decreasing the level of serum osteoblastic marker TRAP in de‐ovulated rats [60]. Ferulic acid is also an active ingredient in Radix Angelicae Sinensis and was found to play a role in inhibiting glucocorticoid‐induced bone loss by increasing the expression level of SIRT1 and decreasing the expression level of NF‐*κ*B in a study by Hou et al. [60]. On the other hand, ferulic acid was found to induce bone loss by upregulating the mRNA expression levels of cyclin D1 and vascular endothelial growth factor (VEGF), and thus inducing bone formation by upregulating the mRNA expression levels of VEGF and SIRT1 in a study by Wang et al. [62]. In a study by Lim et al. [63], *A. sinensis* Diels extract was found to inhibit bone loss due to bone turnover by suppressing bone turnover markers such as serum ALP, CTx and OC levels without affecting oestrogen levels and without any toxicity. Although the aforementioned positive effects of TCM on bone metabolism regulation as well as angiogenesis, the potential pharmacological mechanisms of SND drugs for the treatment of NONFH remain unclear and need to be further explored.

We used UHPLC‐Q‐TOF‐MS^E^ technology combined with UNIFI platform to identify the whole components of SND, and obtained a total of 127 chemical components, while the cleavage patterns of flavonoids, phenols and other compounds were summarised. Based on the CHM similarity theory, 47 active ingredients of SND were screened in the SwissADME platform, and their related targets were predicted in Swiss Target Prediction and other databases to construct a SND target dataset; at the same time, a NONFH target set was obtained and established based on the GSE123568 dataset, and 46 candidate targets were obtained by taking the intersection of the two. KEGG enrichment analysis showed that the candidate targets were mainly involved in NETs formation, HIF‐1 signalling pathway, chemokine signalling pathway, NOD‐like receptor signalling pathway and other signalling pathways. Signalling pathway, chemokine signalling pathway, NOD‐like receptor signalling pathway and other signalling pathways, most of which are related to the innate or reactive immune system, confirming the key role of the immune system in NONFH.

In recent years, immune regulation and its role in the bone tissue immune environment have become a focal point of research. Studies have demonstrated a close interplay between immune dysfunction and bone homeostasis, which is associated with pathological changes in bone tissue [64–66]. In this context, neutrophils play a significant role, particularly through the formation of NETs via NETosis, a critical phenotype in bone immune regulation [65]. NETosis is a mesh‐like structure secreted by activated neutrophils, primarily composed of DNA, histones and various antimicrobial proteins [67]. This structure is involved in pathological processes through multiple mechanisms, including inflammation, procoagulant activity and organ damage. Notably, specific triggers, such as glucocorticoids or alcohol, can activate neutrophils, prompting the release of NETs [18, 68]. The histones and proteases (e.g. myeloperoxidase and neutrophil elastase) within NETs can directly damage endothelial cells, provoke local inflammatory responses and exacerbate ischaemia. In a study by Nonokawa et al. [49] it was confirmed that the development of ONFH is closely associated with the formation of NETs in the small vessels surrounding the lesion. This finding further highlights the significant role of NETs in bone tissue pathogenesis. Similarly, hypoxia is considered one of the primary pathological and physiological characteristics of bone necrosis [69]. HIF‐1*α* is activated under hypoxic conditions and serves as a key regulator in response to hypoxia [70]. The activation of HIF‐1*α* enhances the expression of downstream target genes critical for hypoxia adaptation, including transforming growth factor‐*β* (TGF‐*β*), VEGF and stromal cell‐derived factor‐1 (SDF‐1) [71, 72]. The HIF‐*α* pathway acts as the central regulator of the adaptive response to low oxygen levels and plays a key role in angiogenesis‐osteogenesis coupling [73]. Research has shown that HIF‐1*α* not only promotes osteoclast formation and enhances their activity but also that its downstream target VEGF is essential for maintaining bone homeostasis and promoting skeletal development [74]. Recent studies have further demonstrated that the injection of neutrophils capable of forming NETs into rats can lead to their migration to the surrounding tissues of the femoral head, causing local ischaemia and increasing the expression of HIF‐1*α* in the tissue [75]. This discovery sheds light on the potential mechanism of NETs in the pathological process of bone necrosis. Similarly, hypoxia is considered a primary pathological and physiological characteristic of bone necrosis [49]. HIF‐1*α*, under hypoxic conditions, promotes the division of endothelial progenitor cells into endothelial cells, making it a critical regulator of hypoxia [71]. The activation of HIF‐1*α* strengthens the expression of essential downstream target genes, such as TGF‐*β*, VEGF and SDF‐1 [72, 73]. The HIF‐*α* pathway is the central regulator of the adaptive response to low oxygen levels and plays a key role in angiogenesis‐osteogenesis coupling [74]. Research has shown that HIF‐1*α* promotes osteoclast formation and enhances their activity, while its downstream target VEGF is crucial for maintaining bone homeostasis and promoting skeletal development [75]. Recent studies have demonstrated that the injection of neutrophils capable of forming NETs into rats can lead to their migration to the surrounding tissues of the femoral head, causing local ischaemia and increasing the expression of HIF‐1*α* in the tissue [49].

SVM was identified as the machine learning model with the best predictive performance among the eight constructed models (AUC = 0.778). SHAP analysis, ROC curves and other methods confirmed ACP1, NDUFAF3, HCK, CXCR2 and PTAFR as key targets. These proteins are potential biomarkers involved in the pathogenesis of ONFH. HCK is a key member of the Src family of tyrosine kinases and is widely expressed in neutrophils and osteoclasts, among other myeloid cells, where it plays a critical regulatory role [76]. In neutrophils, HCK participates in pathways involving G protein‐coupled receptors (GPCRs), Fc*γ* receptors and *β*2 integrins, regulating chemotaxis, degranulation, reactive oxygen species (ROS) generation and inflammatory responses. It serves as a pivotal signalling node for neutrophils in their immune defence functions [77]. In osteoclasts, HCK expression is upregulated in response to RANKL stimulation, promoting osteoclast differentiation and activation of the autophagy‐lysosome pathway, thereby enhancing their ability to degrade bone matrix [78–81]. Studies have shown that HCK deficiency significantly inhibits osteoclast formation and function, alleviating bone loss caused by oestrogen deficiency. In both neutrophils and osteoclasts, HCK exerts its effects by activating downstream signalling modules such as Syk, PI3K, PLC*γ*2 and Vav, reflecting the shared mechanistic roles of myeloid cells in immune regulation and bone metabolism [77]. Therefore, HCK is a critical regulator of neutrophil immune responses and osteoclast bone resorption, and it may also serve as a therapeutic target for treating inflammatory diseases and bone metabolic disorders. PTAFR, a membrane protein, specifically binds to platelet‐activating factor (PAF) and mediates various BPs, including immune responses, inflammation, platelet aggregation and vascular regulation, thereby contributing to the progression of diseases such as asthma, cardiovascular disorders and cancer [82–84]. Most researchers agree that the pathogenesis of glucocorticoid‐induced bone disease involves increased apoptosis of osteoblasts and osteocytes, as well as prolonged osteoclast lifespan. Excessive glucocorticoid use not only increases ROS levels but also alters the sensitivity of vascular cells to vasoactive substances, leading to endothelial cell apoptosis [85]. In a study by Hikiji and colleagues, it was found that PTAFR is highly expressed in osteoclasts and is activated through autocrine and paracrine mechanisms, thereby enhancing their responsiveness to inflammatory cytokines such as TNF‐*α* and IL‐1*β*. This activation prolongs the lifespan of osteoclasts and increases their calcium resorption activity, thereby accelerating bone loss. Oestrogen deficiency further upregulates inflammatory cytokines, amplifying PTAFR‐mediated signalling and exacerbating bone resorption. In contrast, PTAFR deficiency or the use of PTAFR antagonists significantly reduces bone loss in ovariectomised mice, suggesting that PTAFR acts as a bridge connecting inflammation and bone metabolic disorders [86]. ACP1 encodes a low molecular weight protein tyrosine phosphatase that regulates protein tyrosine phosphorylation states and participates in bone cell signalling. It plays a significant role in maintaining bone and lipid metabolic homeostasis [87, 88]. Research has shown that certain polymorphisms in the ACP1 gene are associated with an increased risk of bone necrosis in children undergoing treatment for acute lymphoblastic leukaemia, indicating a potential role for ACP1 in the pathogenesis of bone necrosis [89, 90]. ACP1 exerts its effects through its phosphatase activity, regulating multiple signalling pathways that influence bone cell function and bone tissue stability. Genetic variations in ACP1 may serve as potential biomarkers for predicting the risk of hormone‐related bone necrosis [91–93]. CXCR2 is a G protein‐coupled receptor that primarily mediates neutrophil chemotaxis and inflammatory responses. Recent studies have highlighted its important roles in bone metabolism and tissue necrosis [94–96]. In bone metabolism, CXCR2 promotes endothelial cell migration and angiogenesis by activating the Src‐MAP4K4‐p38 MAPK signalling pathway, thereby supporting osteoblast function and bone tissue remodelling [97]. In addition, it collaborates with CXCR4 to regulate the mobilisation of neutrophils from the bone marrow to peripheral tissues, maintaining the stability of the bone marrow microenvironment [96]. In the context of tissue necrosis, CXCR2 deficiency results in abnormal neutrophil accumulation, which triggers inflammation and tissue necrosis. This suggests that CXCR2 plays a protective role in preventing uncontrolled inflammation [98, 99]. Therefore, CXCR2 and its downstream signalling pathways may represent potential therapeutic targets for regulating bone repair and preventing tissue necrosis. NDUFAF3 is a critical assembly factor for mitochondrial Complex I, playing a vital role in maintaining mitochondrial function and cellular energy metabolism [100–102]. In bone metabolism, NDUFAF3 may regulate energy supply to osteoblasts and osteoclasts, influence ROS levels and induce oxidative stress and apoptosis. Additionally, dysfunction of NDUFAF3 may lead to mitochondrial damage and inflammation, thereby promoting bone cell death and the progression of bone necrosis [103, 104]. Although direct evidence is limited, the potential connection between NDUFAF3 and bone remodelling warrants further investigation, as it may offer new therapeutic targets for bone metabolic diseases.

Finally, we used AutoDockTools 1.5.6 and Autodock Vina to validate the MD of the above five key targets and their corresponding active ingredients. The results showed that there was a tight binding relationship between HCK and chrysophanic acid (−8.7 kcal/mol). The MD simulation results suggested a high stability and a satisfactory binding strengthd. Chrysophanic acid is a natural anthraquinone compound derived from Achyranthis Bidentatae Radix, which has many beneficial pharmacological activities such as lipid metabolism regulation, anti‐inflammatory, hepatoprotective, neuroprotective, and so on [105, 106]. In the study of Yu et al. [107], chrysophanic acid was able to promote osteogenic differentiation of BMSCs by up‐regulating the levels of osteogenic markers such as OCN, RUNX2 and OPN, and improve the radiological and histological characteristics of rat femoral head to act as an anti‐NONFH. In addition, chrysophanic acid could regulate lipid homeostasis and cholesterol levels by mediating the AMPK pathway and chrysophanic acid can reverse p‐AMPK levels reduced by alcohol [94]. Senkyunolide F, identified in Angelicae Sinensis Radix, belongs to the class of phenylphthalides, and fewer studies have been conducted on the role and mechanism of this compound [108]. Quillaic acid, also identified in Achyranthis Bidentatae Radix, belongs to the pentacyclic triterpenoids. This compound exhibits various pharmacological activities, including haemolytic, anti‐inflammatory, immunostimulatory, anti‐injurious, antiviral and cytotoxic effects [109].

Notwithstanding the systematic approach and promising insights of this study, several limitations must be acknowledged. Firstly, the identification of the core herbal combination (SND) through data mining of clinical literature carries an inherent risk of selection bias. Secondly, the pharmacological networks and key targets constructed are predominantly based on in silico predictions and bioinformatic analyses; although substantiated by MD and MD simulations, these findings remain hypothetical and require direct experimental confirmation. Thirdly, whilst the UHPLC‐Q‐TOF‐MS^E^ based component identification, supported by comparison with reference standards, has reliably characterised numerous constituents, the complex nature of the herbal matrix means the identification of some compounds still relies on database matching and fragmentation pattern interpretation; thus, their accuracy warrants further verification with additional specific reference standards. Finally, the immune infiltration analysis utilised transcriptomic data from peripheral blood samples, which may not fully recapitulate the precise immune cell composition and interactions within the local bone marrow and necrotic femoral head tissue microenvironment. Consequently, future research should prioritise experimental validation, encompassing: in vitro assays to verify the effects of core active compounds (e.g. Chrysophanic acid on HCK activity, NETosis and osteoclast differentiation); in vivo efficacy studies in established NONFH animal models to evaluate the therapeutic effect of SND and its impact on the identified immunological pathways and key targets within femoral head tissue; and further pharmacokinetic studies to elucidate the bioavailability and tissue distribution of SND’s active components. Addressing these limitations is paramount to translating the findings of this research into clinically actionable knowledge.

## 5. Conclusion

In this study, the active ingredients and potential targets of SND for the treatment of NONFH were investigated and analysed through data mining and literature search, and 47 active ingredients and 46 candidate targets were identified. Correlation analysis was performed on the functions of the candidate targets, and 31 pathways were screened by GO and KEGG enrichment analysis, and NETs formation was identified as the pathway with the lowest *p*‐value and was significantly involved in the treatment of NONFH by SND. In addition, five key targets, namely, ACP1, NDUFAF3, HCK, CXCR2 and PTAFR, were identified in this study by constructing a machine learning model, and the binding activities of the key targets with the corresponding components were verified using MD and kinetic simulation.

In conclusion, this study systematically explored the mechanisms by integrating data mining techniques, UHPLC‐Q‐TOF‐MS^E^ analytical methods, targeted pharmacological studies and MD techniques. This study aims to provide a referenceable research method for subsequent academic research and to provide a specific theoretical basis and practical reference for the clinical treatment of NONFH, with a view to promoting the further development of this field.

## Consent

The authors have nothing to report.

## Disclosure

All authors have read and approved the manuscript.

## Conflicts of Interest

The authors declare no conflicts of interest.

## Author Contributions

Yu Zhou, Wei Huang and Yusong Liu designed the study. Xin Li drafted the manuscript. Xin Li, Liqi Ng, Leilei Qin, Qiuping Zhang, Caiying Liu and Pengcheng Xiao collected the data and analysed the study. Chaozong Liu, Liqi Ng, Yusong Liu and Wei Huang reviewed and edited the manuscript. Yu Zhou and Wei Huang contributed equally to this study.

## Funding

This study was funnded by the Chongqing Traditional Chinese Medical Scientific Research Project (Joint project of Chongqing Health Commission and Science and Technology Bureau) (Grant 2022ZDXM038) and the Tri‐Platform Distinguished Teacher Capacity Enhancement Project of Chongqing University of Chinese Medicine (Grant SOMS20240NXM‐013).

## Supporting Information

Additional supporting information can be found online in the Supporting Information section.

## Supporting information


**Supporting Information** Table S1. Data mining identifies six novel potential formulation combinations. Table S2. 40 medicinal combination patterns derived using the Apriori association rule algorithm. Table S3. 47 chemical constituents in SND complying with drug‐likeness screening rules. Table S4. 46 targets at the cross‐talk between NONFH and SND. Table S5. GO/KEGG enrichment analysis results of the cross‐targets between five NONFH–SND. Table S6. The binding energies between five key targets and their corresponding compounds.

## Data Availability

The datasets used and/or analysed during the current study are available from the corresponding author upon reasonable request.
