# Supplementary material for: Immunomodulatory Mechanisms of Rehmanniae Radix Praeparata–Achyranthes Root–Chinese Angelica Root Combination in Nontraumatic Osteonecrosis of the Femoral Head: A Comprehensive Network Pharmacology and Molecular Docking Study Focusing on Immunological Pathways
Source: Mediators Inflamm. 2025 Dec 20;2025:2808908. doi: 10.1155/mi/2808908 (PMC12767418; doi:10.1155/mi/2808908)
Supplement: Supplementary file 1 — Supporting Information Table S1. Data mining identifies six novel potential formulation combinations. Table S2. 40 medicinal combination patterns derived using the Apriori association rule algorithm. Table S3. 47 chemical constituents in SND complying with drug‐likeness screening rules. Table S4. 46 targets at the cross‐talk between NONFH and SND. Table S5. GO/KEGG enrichment analysis results of the cross‐targets between five NONFH–SND. Table S6. The binding energies between five key targets and their corresponding compounds. [file MI-2025-2808908-s001.docx]

**Supplementary Table 1** Data Mining Identifies 6 Novel Potential Formulation Combinations

| NO. | New formula combination |
| --- | --- |
| **0** | Cinnamomi Cortex_Scorpio_Hirudo_Spatholobi Caulis_Dipsaci Radix |
| **1** | Cinnamomi Cortex_Corydalis Rhizoma_Notoginseng Radix Et Rhizoma_Poria_Alismatis Rhizoma |
| **2** | Taxilli Herba_Epimedii Folium_Alismatis Rhizoma_Cuscutae Semen |
| **3** | Poria_Glycyrrhizae Radix Et Rhizoma_Atractylodis Macrocephalae Rhizoma_Corni Fructus_Cervi Cornus Colla |
| **4** | Atractylodis Rhizoma_Angelicae Sinensis Radix_Rehmanniae Radix Praeparata_Astragali Radix_Salviae Miltiorrhizae Radix Et Rhizoma_Cervi Cornu Pantotrichum |
| **5** | Rehmanniae Radix_Dioscoreae Rhizoma_Psoraleae Fructus_Notoginseng Radix Et Rhizoma |

**Supplementary Table 2** 40 Medicinal Combination Patterns Derived Using the Apriori Association Rule Algorithm

| **NO.** | CHMs combination mode | Frequency | **NO.** | CHMs combination mode | Frequency |
| --- | --- | --- | --- | --- | --- |
| **1** | Rehmanniae Radix Praeparata,Chuanxiong Rhizoma | 16 | **21** | Achyranthis Bidentatae Radix,Poria | 19 |
| **2** | Rehmanniae Radix Praeparata,Angelicae Sinensis Radix | 21 | **22** | Salviae Miltiorrhizae Radix Et Rhizoma,Drynariae Rhizoma | 15 |
| **3** | Rehmanniae Radix Praeparata,Achyranthis Bidentatae Radix | 18 | **23** | Drynariae Rhizoma,Glycyrrhizae Radix Et Rhizoma | 19 |
| **4** | Rehmanniae Radix Praeparata,Drynariae Rhizoma | 15 | **24** | Salviae Miltiorrhizae Radix Et Rhizoma,Glycyrrhizae Radix Et Rhizoma | 17 |
| **5** | Chuanxiong Rhizoma,Angelicae Sinensis Radix | 20 | **25** | Glycyrrhizae Radix Et Rhizoma,Dipsaci Radix | 16 |
| **6** | Chuanxiong Rhizoma,Achyranthis Bidentatae Radix | 15 | **26** | Atractylodis Macrocephalae Rhizoma,Glycyrrhizae Radix Et Rhizoma | 21 |
| **7** | Glycyrrhizae Radix Et Rhizoma,Epimedii Folium | 15 | **27** | Atractylodis Macrocephalae Rhizoma,Poria | 18 |
| **8** | Achyranthis Bidentatae Radix,Angelicae Sinensis Radix | 24 | **28** | Glycyrrhizae Radix Et Rhizoma,Poria | 20 |
| **9** | Drynariae Rhizoma,Angelicae Sinensis Radix | 22 | **29** | Rehmanniae Radix Praeparata,Achyranthis Bidentatae Radix,Angelicae Sinensis Radix | 17 |
| **10** | Salviae Miltiorrhizae Radix Et Rhizoma,Angelicae Sinensis Radix | 17 | **30** | Achyranthis Bidentatae Radix,Drynariae Rhizoma,Angelicae Sinensis Radix | 17 |
| **11** | Dipsaci Radix,Angelicae Sinensis Radix | 18 | **31** | Achyranthis Bidentatae Radix,Glycyrrhizae Radix Et Rhizoma,Angelicae Sinensis Radix | 18 |
| **12** | Atractylodis Macrocephalae Rhizoma,Angelicae Sinensis Radix | 17 | **32** | Achyranthis Bidentatae Radix,Angelicae Sinensis Radix,Poria | 15 |
| **13** | Glycyrrhizae Radix Et Rhizoma,Angelicae Sinensis Radix | 25 | **33** | Atractylodis Macrocephalae Rhizoma,Glycyrrhizae Radix Et Rhizoma,Angelicae Sinensis Radix | 16 |
| **14** | Angelicae Sinensis Radix,Poria | 19 | **34** | Atractylodis Macrocephalae Rhizoma,Angelicae Sinensis Radix,Poria | 15 |
| **15** | Eucommiae Cortex,Angelicae Sinensis Radix | 15 | **35** | Glycyrrhizae Radix Et Rhizoma,Angelicae Sinensis Radix,Poria | 17 |
| **16** | Achyranthis Bidentatae Radix,Drynariae Rhizoma | 23 | **36** | Achyranthis Bidentatae Radix,Drynariae Rhizoma,Glycyrrhizae Radix Et Rhizoma | 16 |
| **17** | Salviae Miltiorrhizae Radix Et Rhizoma,Achyranthis Bidentatae Radix | 17 | **37** | Achyranthis Bidentatae Radix,Atractylodis Macrocephalae Rhizoma,Glycyrrhizae Radix Et Rhizoma | 16 |
| **18** | Achyranthis Bidentatae Radix,Dipsaci Radix | 16 | **38** | Achyranthis Bidentatae Radix,Atractylodis Macrocephalae Rhizoma,Poria | 15 |
| **19** | Achyranthis Bidentatae Radix,Atractylodis Macrocephalae Rhizoma | 19 | **39** | Achyranthis Bidentatae Radix,Glycyrrhizae Radix Et Rhizoma,Poria | 17 |
| **20** | Achyranthis Bidentatae Radix,Glycyrrhizae Radix Et Rhizoma | 24 | **40** | Atractylodis Macrocephalae Rhizoma,Glycyrrhizae Radix Et Rhizoma,Poria | 16 |

**Supplementary Table 3** 47 Chemical Constituents in SND Complying with Drug-Likeness Screening Rules

| **NO.** | Drug mode | **NO.** | Drug mode |
| --- | --- | --- | --- |
| **1** | 1,2,3,4-Tetrahydroharmane-3-carboxylic acid | **25** | Baicalein |
| **2** | Phthalic acid | **26** | Chrysophanic acid |
| **3** | Vanillic acid | **27** | pinellic acid |
| **4** | Dihydrocaffeic acid | **28** | Epinortrachelogenin |
| **5** | Senkyunolide R | **29** | Coniferyl ferulate |
| **6** | Ecdysterone | **30** | Nobiletin |
| **7** | Rehmapicrogenin | **31** | 23-hydroxytormentic acid |
| **8** | Ferulic acid | **32** | Lauryldiethanolamine |
| **9** | makisterone B | **33** | Dibutyl phthalate |
| **10** | Acetovanillone | **34** | 3-Butylidenephthalide |
| **11** | N-acetyltryptophan | **35** | 9,10-dihydroxy-12Z-octadecenoic acid |
| **12** | Azelaic acid | **36** | 4-hydroxy3-butylphthalide |
| **13** | Senkyunolide S | **37** | Senkyunolide K |
| **14** | marmesin | **38** | Ligustilide |
| **15** | Senkyunolide F | **39** | Tokinolide B |
| **16** | Formononetin | **40** | Isoferulic acid |
| **17** | N-Trans-Feruloyltyramine | **41** | Quillaic acid |
| **18** | Butylparaben | **42** | 13-hydroxy-9,11-octadecadienoic acid |
| **19** | N-Feruloyl-3-methoxytyramine | **43** | inokosterone |
| **20** | 6-methoxycoumarin | **44** | 13-keto-9Z,11E-octadecadienoic acid |
| **21** | Kaempferol | **45** | 3-n-Butylphthalide |
| **22** | Wogonin | **46** | CORTEXOLONE |
| **23** | Malic acid | **47** | Octadecanamide |
| **24** | (10E,15E)-9,12,13-trihydroxyoctadeca- 10,  15-dienoic acid | **-** | - |

**Supplementary Table 4** 46 Targets at the Cross-Talk Between NONFH and SND

| **NO.** | **Target** | **NO.** | **Target** |
| --- | --- | --- | --- |
| 1 | EGLN1 | 24 | IGF2R |
| 2 | MAP3K5 | 25 | NDUFAF3 |
| 3 | FPR2 | 26 | PDK3 |
| 4 | ELF4 | 27 | PTPN12 |
| 5 | CCR1 | 28 | LRRK2 |
| 6 | TLR4 | 29 | TBXAS1 |
| 7 | C5AR1 | 30 | CASP1 |
| 8 | PRKCD | 31 | CASP4 |
| 9 | PKN2 | 32 | CXCR2 |
| 10 | TLR8 | 33 | PTAFR |
| 11 | SLC8A1 | 34 | ALDH5A1 |
| 12 | PTGS2 | 35 | NLRP3 |
| 13 | NAMPT | 36 | ASAH1 |
| 14 | SELL | 37 | RPS6KA5 |
| 15 | LPAR2 | 38 | MAP3K8 |
| 16 | TLR2 | 39 | LYN |
| 17 | ACP1 | 40 | ITM2B |
| 18 | SOAT1 | 41 | CXCR1 |
| 19 | OGFRL1 | 42 | MAP3K3 |
| 20 | ABCG2 | 43 | MPEG1 |
| 21 | SLC7A5 | 44 | CTSS |
| 22 | TLR1 | 45 | FFAR2 |
| 23 | RAF1 | 46 | HCK |

**Supplementary Table 5** GO/KEGG enrichment analysis results of the cross-targets between 5 NONFH-SND

| Category | Term | Count | % | *P*-Value | Genes | List Total | Pop Hits | Pop Total | Fold Enrichment | Bonferroni | Benjamini | FDR |
| --- | --- | --- | --- | --- | --- | --- | --- | --- | --- | --- | --- | --- |
| KEGG_PATHWAY | hsa04613:Neutrophil extracellular trap formation | 8 | 17.391304347826086 | 2.1088221853428934E-5 | CASP4, C5AR1, CASP1, TLR8, FPR2, RAF1, TLR4, TLR2 | 41 | 193 | 8865 | 8.962466826740807 | 0.0036416538851563596 | 0.0036271741587897764 | 0.0033741154965486295 |
| KEGG_PATHWAY | hsa04621:NOD-like receptor signaling pathway | 7 | 15.217391304347828 | 1.8208365510487658E-4 | CASP4, NAMPT, PRKCD, CASP1, NLRP3, PKN2, TLR4 | 41 | 189 | 8865 | 8.008130081300814 | 0.031012280495730016 | 0.011701186947435518 | 0.010884825067381877 |
| KEGG_PATHWAY | hsa04062:Chemokine signaling pathway | 7 | 15.217391304347828 | 2.040904700134102E-4 | LYN, CCR1, HCK, CXCR1, CXCR2, PRKCD, RAF1 | 41 | 193 | 8865 | 7.8421584733982055 | 0.0346950862751817 | 0.011701186947435518 | 0.010884825067381877 |
| UP_TISSUE | Monocyte | 4 | 8.695652173913043 | 3.781502144617375E-4 | CCR1, FPR2, TLR4, TLR2 | 45 | 59 | 18393 | 27.710734463276836 | 0.0309047780921079 | 0.03176461801478595 | 0.029873866942477262 |
| UP_TISSUE | Placenta | 19 | 41.30434782608695 | 0.0012002205527003166 | LYN, MAP3K3, PTAFR, PTGS2, CTSS, SLC8A1, SLC7A5, RPS6KA5, SOAT1, CXCR1, ELF4, CASP4, CXCR2, TLR8, PKN2, RAF1, ACP1, TLR4, ABCG2 | 45 | 3679 | 18393 | 2.1108815801395306 | 0.0948713002464876 | 0.0504092632134133 | 0.04740871183166251 |
| KEGG_PATHWAY | hsa04625:C-type lectin receptor signaling pathway | 5 | 10.869565217391305 | 0.0012236965762012677 | PRKCD, CASP1, NLRP3, RAF1, PTGS2 | 41 | 105 | 8865 | 10.296167247386759 | 0.19089707207341988 | 0.048359894607434756 | 0.044985948472032335 |
| KEGG_PATHWAY | hsa04620:Toll-like receptor signaling pathway | 5 | 10.869565217391305 | 0.0014058108897510105 | TLR1, TLR8, MAP3K8, TLR4, TLR2 | 41 | 109 | 8865 | 9.918326247482659 | 0.2160236262668087 | 0.048359894607434756 | 0.044985948472032335 |
| KEGG_PATHWAY | hsa04722:Neurotrophin signaling pathway | 5 | 10.869565217391305 | 0.002003163547086293 | MAP3K3, RPS6KA5, PRKCD, RAF1, MAP3K5 | 41 | 120 | 8865 | 9.009146341463415 | 0.29312034960297595 | 0.057424021683140404 | 0.053417694588967814 |
| KEGG_PATHWAY | hsa05417:Lipid and atherosclerosis | 6 | 13.043478260869565 | 0.002693585864746569 | LYN, CASP1, NLRP3, TLR4, TLR2, MAP3K5 | 41 | 216 | 8865 | 6.0060975609756095 | 0.3728810706686053 | 0.06618525267662999 | 0.06156767690849301 |
| KEGG_PATHWAY | hsa05171:Coronavirus disease - COVID-19 | 6 | 13.043478260869565 | 0.004086826111213445 | C5AR1, CASP1, NLRP3, TLR8, TLR4, TLR2 | 41 | 238 | 8865 | 5.450912072145932 | 0.5076028148958038 | 0.08786676139108907 | 0.08173652222426889 |
| KEGG_PATHWAY | hsa05131:Shigellosis | 6 | 13.043478260869565 | 0.0050343467109788655 | RPS6KA5, CASP4, PRKCD, CASP1, NLRP3, TLR4 | 41 | 250 | 8865 | 5.189268292682926 | 0.5823625965381379 | 0.08806059942916157 | 0.08191683667828983 |
| KEGG_PATHWAY | hsa05132:Salmonella infection | 6 | 13.043478260869565 | 0.005119802292393115 | CASP4, CASP1, NLRP3, RAF1, TLR4, TLR2 | 41 | 251 | 8865 | 5.168593917014867 | 0.5885224989836112 | 0.08806059942916157 | 0.08191683667828983 |
| UP_TISSUE | T-cell | 5 | 10.869565217391305 | 0.00537948245859599 | TLR1, CASP4, MAP3K8, IGF2R, TLR2 | 45 | 296 | 18393 | 6.9042792792792795 | 0.36090510493933725 | 0.14167779119534477 | 0.13324458933847902 |
| KEGG_PATHWAY | hsa05235:PD-L1 expression and PD-1 checkpoint pathway in cancer | 4 | 8.695652173913043 | 0.007622981567135982 | MAP3K3, RAF1, TLR4, TLR2 | 41 | 90 | 8865 | 9.609756097560977 | 0.7338853384247144 | 0.11919571177703536 | 0.11087973188561429 |
| UP_TISSUE | Platelet | 6 | 13.043478260869565 | 0.00813020917212948 | LYN, ASAH1, TBXAS1, PRKCD, PTPN12, MAP3K5 | 45 | 526 | 18393 | 4.662357414448669 | 0.4921481375928686 | 0.14167779119534477 | 0.13324458933847902 |
| UP_TISSUE | Blood | 7 | 15.217391304347828 | 0.00843320185686576 | CCR1, CXCR1, NAMPT, FPR2, ACP1, ABCG2, TLR2 | 45 | 749 | 18393 | 3.81993769470405 | 0.5048645350197563 | 0.14167779119534477 | 0.13324458933847902 |
| KEGG_PATHWAY | hsa05152:Tuberculosis | 5 | 10.869565217391305 | 0.008827111962279682 | TLR1, RAF1, TLR4, CTSS, TLR2 | 41 | 182 | 8865 | 5.940096488876976 | 0.7843007484194142 | 0.1265219381260088 | 0.11769482616372909 |
| KEGG_PATHWAY | hsa04666:Fc gamma R-mediated phagocytosis | 4 | 8.695652173913043 | 0.009897575407203317 | LYN, HCK, PRKCD, RAF1 | 41 | 99 | 8865 | 8.736141906873614 | 0.8210789424204509 | 0.13095253615684388 | 0.12181631270404082 |
| UP_TISSUE | Liver | 21 | 45.65217391304348 | 0.01136195739196942 | LYN, ASAH1, LRRK2, PRKCD, PTPN12, CTSS, IGF2R, TLR1, SLC7A5, HCK, ALDH5A1, TBXAS1, NAMPT, PDK3, TLR8, NDUFAF3, MAP3K8, PKN2, RAF1, ACP1, MAP3K5 | 45 | 5209 | 18393 | 1.6478018813591861 | 0.6126554984524164 | 0.15906740348757187 | 0.14959910566093068 |
| KEGG_PATHWAY | hsa05167:Kaposi sarcoma-associated herpesvirus infection | 5 | 10.869565217391305 | 0.011376354404625809 | LYN, CCR1, HCK, RAF1, PTGS2 | 41 | 196 | 8865 | 5.515803882528621 | 0.8618465013314922 | 0.13976663982825993 | 0.13001547891000925 |
| KEGG_PATHWAY | hsa05130:Pathogenic Escherichia coli infection | 5 | 10.869565217391305 | 0.012812663057502742 | CASP4, CASP1, LPAR2, NLRP3, TLR4 | 41 | 203 | 8865 | 5.325603748648324 | 0.8925697411252346 | 0.1469185363926981 | 0.1366684059466959 |
| UP_TISSUE | Granulocyte | 2 | 4.3478260869565215 | 0.01426964761212675 | PTAFR, FPR2 | 45 | 6 | 18393 | 136.24444444444444 | 0.6966634485632556 | 0.171235771345521 | 0.16104316590828763 |
| KEGG_PATHWAY | hsa04668:TNF signaling pathway | 4 | 8.695652173913043 | 0.016242284481260298 | RPS6KA5, MAP3K8, PTGS2, MAP3K5 | 41 | 119 | 8865 | 7.267882762861243 | 0.9411630418981605 | 0.1746045581735482 | 0.162422844812603 |
| UP_TISSUE | Spleen | 7 | 15.217391304347828 | 0.017898196559978603 | HCK, TBXAS1, PTAFR, CASP1, PKN2, TLR4, MPEG1 | 45 | 883 | 18393 | 3.2402416006040013 | 0.7766478132043624 | 0.18793106387977535 | 0.1767446910297887 |
| KEGG_PATHWAY | hsa05135:Yersinia infection | 4 | 8.695652173913043 | 0.0239730990637741 | CASP1, NLRP3, PKN2, TLR4 | 41 | 138 | 8865 | 6.267232237539767 | 0.9849724121776773 | 0.2425513552334791 | 0.22562916765905033 |
| KEGG_PATHWAY | hsa05134:Legionellosis | 3 | 6.521739130434782 | 0.026224413410764067 | CASP1, TLR4, TLR2 | 41 | 56 | 8865 | 11.583188153310102 | 0.9899217153381252 | 0.2505888392584122 | 0.2331058969845695 |
| KEGG_PATHWAY | hsa04072:Phospholipase D signaling pathway | 4 | 8.695652173913043 | 0.029218127230447553 | CXCR1, CXCR2, LPAR2, RAF1 | 41 | 149 | 8865 | 5.804550662956293 | 0.9940837451341857 | 0.26450094124405155 | 0.24604738720376887 |
| UP_TISSUE | Thymus | 6 | 13.043478260869565 | 0.03350495027988281 | SELL, TBXAS1, CASP4, PTPN12, RAF1, MPEG1 | 45 | 756 | 18393 | 3.243915343915344 | 0.9409024306614023 | 0.31271286927890624 | 0.2940990080123047 |
| KEGG_PATHWAY | hsa05120:Epithelial cell signaling in Helicobacter pylori infection | 3 | 6.521739130434782 | 0.04056839373517835 | LYN, CXCR1, CXCR2 | 41 | 71 | 8865 | 9.136035726554448 | 0.9992265617125689 | 0.3488881861225338 | 0.3245471498814268 |
| KEGG_PATHWAY | hsa05164:Influenza A | 4 | 8.695652173913043 | 0.0426065585143272 | CASP1, NLRP3, RAF1, TLR4 | 41 | 173 | 8865 | 4.999295079655998 | 0.9994646380312654 | 0.34896800306972753 | 0.3246213982043977 |
| KEGG_PATHWAY | hsa04010:MAPK signaling pathway | 5 | 10.869565217391305 | 0.045213006156786036 | MAP3K3, RPS6KA5, MAP3K8, RAF1, MAP3K5 | 41 | 300 | 8865 | 3.6036585365853657 | 0.999665940728193 | 0.35247763910855534 | 0.3278861759149352 |
| KEGG_PATHWAY | hsa05133:Pertussis | 3 | 6.521739130434782 | 0.04807220523033372 | CASP1, NLRP3, TLR4 | 41 | 78 | 8865 | 8.316135084427767 | 0.9998011647710114 | 0.35247763910855534 | 0.3278861759149352 |
| KEGG_PATHWAY | hsa05140:Leishmaniasis | 3 | 6.521739130434782 | 0.049182926387240276 | PTGS2, TLR4, TLR2 | 41 | 79 | 8865 | 8.210867551713491 | 0.999837528909078 | 0.35247763910855534 | 0.3278861759149352 |
| KEGG_PATHWAY | hsa04912:GnRH signaling pathway | 3 | 6.521739130434782 | 0.06567167287954156 | MAP3K3, PRKCD, RAF1 | 41 | 93 | 8865 | 6.974822974036191 | 0.9999921219945265 | 0.451821109411246 | 0.420298706429066 |
| KEGG_PATHWAY | hsa04061:Viral protein interaction with cytokine and cytokine receptor | 3 | 6.521739130434782 | 0.07452080201683273 | CCR1, CXCR1, CXCR2 | 41 | 100 | 8865 | 6.486585365853658 | 0.9999984814382783 | 0.4548783500538831 | 0.4231426512129145 |
| KEGG_PATHWAY | hsa05150:Staphylococcus aureus infection | 3 | 6.521739130434782 | 0.07711675144868932 | C5AR1, PTAFR, FPR2 | 41 | 102 | 8865 | 6.359397417503586 | 0.9999990659072223 | 0.4548783500538831 | 0.4231426512129145 |
| KEGG_PATHWAY | hsa04151:PI3K-Akt signaling pathway | 5 | 10.869565217391305 | 0.07895759496070345 | LPAR2, PKN2, RAF1, TLR4, TLR2 | 41 | 362 | 8865 | 2.986457350761353 | 0.9999993387354446 | 0.4548783500538831 | 0.4231426512129145 |
| UP_TISSUE | Promyelocytic leukemia | 2 | 4.3478260869565215 | 0.08048183635476767 | EGLN1, ELF4 | 45 | 35 | 18393 | 23.356190476190477 | 0.9990548386915536 | 0.6153686101692072 | 0.5787395262305638 |
| UP_TISSUE | Peripheral blood | 3 | 6.521739130434782 | 0.08058398466501522 | SLC7A5, TBXAS1, PTGS2 | 45 | 197 | 18393 | 6.224365482233502 | 0.9990635138671058 | 0.6153686101692072 | 0.5787395262305638 |
| KEGG_PATHWAY | hsa04064:NF-kappa B signaling pathway | 3 | 6.521739130434782 | 0.08106436123179414 | LYN, PTGS2, TLR4 | 41 | 105 | 8865 | 6.177700348432055 | 0.9999995550395343 | 0.4548783500538831 | 0.4231426512129145 |
| KEGG_PATHWAY | hsa05163:Human cytomegalovirus infection | 4 | 8.695652173913043 | 0.08114627263530483 | CCR1, CXCR2, RAF1, PTGS2 | 41 | 226 | 8865 | 3.826894021152601 | 0.999999561848823 | 0.4548783500538831 | 0.4231426512129145 |
| KEGG_PATHWAY | hsa05208:Chemical carcinogenesis - reactive oxygen species | 4 | 8.695652173913043 | 0.08198388867250218 | PRKCD, RAF1, ACP1, MAP3K5 | 41 | 227 | 8865 | 3.810035457182766 | 0.9999996258011449 | 0.4548783500538831 | 0.4231426512129145 |
| UP_TISSUE | Macrophage | 2 | 4.3478260869565215 | 0.08926642248010493 | SOAT1, CASP1 | 45 | 39 | 18393 | 20.96068376068376 | 0.9995739290308473 | 0.6248649573607344 | 0.5876706146606907 |
| UP_TISSUE | Lung | 11 | 23.91304347826087 | 0.0973452046841779 | EGLN1, OGFRL1, SELL, TBXAS1, NAMPT, PTAFR, LPAR2, NDUFAF3, PTGS2, TLR4, MAP3K5 | 45 | 2678 | 18393 | 1.678889718695544 | 0.9997966235912437 | 0.6289997841131495 | 0.591559320773081 |

**Supplementary Table 6** The binding energies between 5 key targets and their corresponding compounds

| **Target** | **Compounds** | **Binding energy(Kcal/mol)** |
| --- | --- | --- |
| **ACP1** | 9,10-dihydroxy-12Z-octadecenoic acid | -5.1 |
|  | 23-hydroxytormentic acid | -7.4 |
|  | Baicalein | -7.2 |
|  | CORTEXOLONE | -7.2 |
|  | Formononetin | -6.6 |
|  | Kaempferol | -7.3 |
|  | Quillaic acid | -7.5 |
|  | Rehmapicrogenin | -5.3 |
| **NDUFAF3** | 6-methoxycoumarin | -4.8 |
|  | Acetovanillone | -4.4 |
|  | Coniferyl ferulate | -5.5 |
|  | Epinortrachelogenin | -5.8 |
|  | Ferulic acid | -4.7 |
|  | N-Feruloyl-3-methoxytyramine | -5.7 |
|  | N-Trans-Feruloyltyramine | -5.9 |
|  | Vanillic acid | -4.7 |
| **CXCR2** | 4-hydroxy3-butylphthalide | -6.4 |
|  | 13-keto-9Z,11E-octadecadienoic acid | -6.4 |
|  | Dibutyl phthalate | -7.3 |
|  | Epinortrachelogenin | -6.8 |
|  | N-acetyltryptophan | -6.1 |
| **HCK** | Chrysophanic acid | -8.7 |
|  | Epinortrachelogenin | -8.3 |
| **PTAFR** | Senkyunolide F | -7.8 |
